# Physicochemical evaluation of pediatric liquid medicines in Ghana: implications for cariogenicity and erosive potential

**DOI:** 10.1186/s12903-026-09011-0

**Published:** 2026-06-27

**Authors:** Gideon Atinga Akolgo, Richard Addo-Owusu, Richard Anthony Opong, Rhema Harry Andoh

**Affiliations:** 1 Department of Biochemistry, Cell and Molecular Biology, College of Basic and Applied Sciences, University of Ghana, Legon, Accra Ghana; 2 Department of Pharmaceutical Chemistry, Entrance University of Health Sciences, Spintex Road, Accra, Ghana

**Keywords:** Pediatric liquid medicines, Dental caries, Tooth erosion, Physicochemical properties, Oral health

## Abstract

**Background:**

Pediatric liquid medicines are indispensable and widely used in childhood disease management, but often contain fermentable sugars and acidic excipients that may increase the risk of dental caries and tooth erosion. This study conducted a physicochemical evaluation of pediatric liquid medicines marketed in Ghana to determine their potential contribution to dental caries and tooth erosion, conditions associated with acidic and sugar-containing formulations.

**Methods:**

Thirty pediatric liquid medicines across six therapeutic classes (analgesics, antitussives, antihistamines, antibiotics, antimalarials, and non-steroidal anti-inflammatory drugs) were analysed. Physicochemical parameters assessed included pH, total titratable acidity (TTA; % citric acid equivalents), sucrose content (%), and total soluble solids content (TSSC; °Brix). All analyses were performed in triplicate, and comparisons across therapeutic classes were conducted using one-way analysis of variance (ANOVA) followed by post hoc multiple comparison tests, with statistical significance set at *p* < 0.05.

**Results:**

The pH of the medicines ranged from 2.76 to 7.57, with 70% below the critical enamel demineralisation threshold (pH 5.5). TTA varied from 0.01% to 1.66%, with antibiotics and non-steroidal anti-inflammatory drugs exhibiting the highest mean values. Sucrose content ranged from 0.0% to 75.6%, with 43.3% of products exceeding the 10% cariogenic threshold. TSSC values ranged from 4.2°Brix to 61.8°Brix, with several formulations exceeding the high-risk threshold of 30°Brix. Comparative analyses revealed no statistically significant differences in pH (*p* = 0.130), TTA (*p* = 0.063), or sucrose content (*p* = 0.952) across therapeutic classes. However, a statistically significant difference was observed for TSSC (*p* = 0.010), indicating variability in overall soluble solid concentration among the therapeutic categories. Antitussives and NSAIDs showed the highest overall erosive potential, while antihistamines and non-steroidal anti-inflammatory drugs demonstrated elevated cariogenic risk. Locally manufactured products generally had higher sucrose content, whereas some imported formulations exhibited higher titratable acidity.

**Conclusions:**

Many pediatric liquid medicines marketed in Ghana exhibit physicochemical properties that may contribute to dental caries and tooth erosion. These findings underscore the need for targeted public health interventions, including regulatory policies to limit sugar and acidity in paediatric formulations, promotion of sugar-free alternatives, and integration of oral health counselling into paediatric prescribing practices, particularly in low- and middle-income settings.

**Supplementary Information:**

The online version contains supplementary material available at 10.1186/s12903-026-09011-0.

## Background

Dental caries and erosive tooth wear remain among the most prevalent chronic oral conditions affecting children worldwide. According to the Global Burden of Disease Study, untreated dental caries in primary teeth affects more than 520 million children globally, representing one of the most common non-communicable diseases in childhood [1]. Despite being largely preventable, caries continues to impose substantial functional, psychosocial, and economic burdens, particularly in low- and middle-income countries (LMICs) where preventive dental services are limited [2]. In parallel, erosive tooth wear, defined as irreversible loss of dental hard tissue due to chemical processes not involving bacterial metabolism, is increasingly recognised as a growing concern in children and adolescents [3].

The pathogenesis of dental caries involves the fermentation of dietary or medicinal sugars by oral microorganisms, leading to acid production and subsequent enamel demineralisation when the plaque pH falls below the critical threshold of approximately 5.5 [4, 5]. Repeated exposure to fermentable carbohydrates promotes ecological shifts in dental biofilm, favouring acidogenic and aciduric species such as *Streptococcus mutans*, thereby accelerating lesion development [6]. In contrast, dental erosion results from direct exposure of tooth surfaces to extrinsic or intrinsic acids, leading to surface softening and mineral loss independent of bacterial activity [3, 7, 8]. Importantly, low pH and high titratable acidity enhance enamel dissolution and prolong demineralisation, especially when salivary buffering capacity is overwhelmed [9–12].

Liquid oral medicines are widely prescribed in pediatric practice due to dosing flexibility, ease of swallowing, and improved patient compliance. Syrups, suspensions, and elixirs are particularly preferred for infants and young children who cannot swallow tablets or capsules. However, to improve palatability, viscosity, and stability, many pediatric liquid formulations contain fermentable sugars (e.g., sucrose, glucose, fructose), viscosifiers, and acidulants (e.g., citric acid, ascorbic acid) [13–18]. These excipients may inadvertently increase cariogenic and erosive risks when medicines are administered frequently or for prolonged periods, particularly in chronically ill children. This is because repeated exposure to sugar-containing, low-pH, and viscous formulations has been shown to decrease enamel microhardness and increase enamel surface loss in vitro, and is associated with higher caries experience in clinical populations requiring long-term medication use [19–21].

Sucrose is of particular concern because it serves as a substrate for glucosyltransferase-mediated synthesis of extracellular polysaccharides, which enhance biofilm adhesion and structural integrity, promoting acid retention and cariogenicity in dental biofilms [22, 23]. The World Health Organization recommends limiting free sugar intake to less than 10% of total energy intake, and ideally below 5%, to reduce the risk of dental caries across the lifespan [24, 25]. Repeated medicinal exposure, especially at night when salivary flow and buffering are reduced, prolongs acidic conditions at the tooth surface and increases cumulative demineralisation risk [26]. Additionally, formulations with low endogenous pH and high titratable acidity have been shown in vitro to exacerbate enamel surface softening and decrease microhardness [27, 28].

Several international studies have characterised the physicochemical properties of pediatric liquid medicines and assessed their potential contribution to dental caries and tooth erosion. For instance, studies across different regions consistently demonstrate substantial variability in the physicochemical characteristics of pediatric liquid medicines, with a high proportion of formulations exhibiting low pH, elevated acidity, and significant sucrose content, all of which are linked to increased cariogenic and erosive risk [28–31]. A recent survey conducted in Turkey reported wide ranges of sucrose content and pH among pediatric syrups, highlighting substantial cariogenic and erosive potential in several commonly used products [32]. In Saudi Arabia, a long-term immersion study demonstrated that pediatric liquid medications with acidic pH and sugar content reduced enamel microhardness, suggesting cumulative erosive effects with prolonged exposure [20]. Additionally, an evaluation of over-the-counter pediatric antipyretics and analgesics reported that many formulations had pH values and buffering capacities consistent with erosive potential, reinforcing the concern that such medicines may pose oral health risks [33]. Collectively, these studies indicate that medicinal formulations and the physicochemical profiles of pediatric liquid medicines, particularly when they contain low pH, high titratable acidity, and substantial sugar may contribute to an under-recognised risk factor for dental demineralisation, especially with frequent or prolonged use.

The evaluation of sugar and acidity in pediatric medicines is also relevant from a pharmaceutical regulatory perspective. International regulatory authorities increasingly recognise the importance of excipient safety in pediatric formulations. Guidance from international regulatory bodies such as the World Health Organization (WHO) and the European Medicines Agency (EMA) recommends careful evaluation of excipients, including sugars, acidifying agents, and sweeteners in pediatric medicines because children may be particularly susceptible to metabolic, toxicological, and dental effects associated with these substances [24, 34, 35]. Similarly, the United States Food and Drug Administration (FDA) and other regulatory guidance documents emphasise careful selection of excipients in pediatric medicines and discourage the use of cariogenic sugars such as sucrose when safer alternatives are available, encouraging the development of sugar-free or non-cariogenic sweetener-based formulations for pediatric oral preparations [36]. Despite these recommendations, regulatory frameworks in many countries focus primarily on therapeutic efficacy and systemic safety, while the oral health implications of excipients receive less attention. Generating physicochemical data on marketed formulations, therefore, provides important evidence to support future regulatory standards and formulation improvements aimed at reducing dental risk in children.

Despite growing global evidence, data from sub-Saharan Africa remain limited. In many African countries, regulatory frameworks focus primarily on therapeutic efficacy and safety, with less emphasis on the dental safety of excipients. In Ghana, pediatric liquid medicines are widely available as both prescription and over-the-counter products through pharmacies and chemical sellers. Patterns of self-medication and limited access to preventive dental care may further increase cumulative exposure risks. However, the physicochemical characteristics of these formulations, including pH, titratable acidity, sugar content, and total soluble solids, have not been systematically evaluated in the Ghanaian context.

Given the high burden of childhood caries in LMICs and increasing availability of pediatric syrups, there is a need to generate context-specific data to inform regulatory standards and encourage reformulation strategies (e.g., sugar-free alternatives, buffering adjustments). Therefore, this study assessed key physicochemical properties of commonly used pediatric liquid medicines in Ghana to estimate their cariogenicity and erosive potential. By providing evidence-based risk profiling, the findings aim to support clinical counselling, public health policy, and pharmaceutical formulation improvements in resource-limited settings.

## Methods

### Study design, sample selection, justification, and risk-scoring framework

#### Study design and sample selection

This study employed a laboratory-based experimental design to evaluate the physicochemical properties and potential oral health risks associated with pediatric liquid medicines marketed in Ghana. The investigation focused on parameters known to influence dental caries and enamel erosion, including pH, titratable acidity, sucrose content, and total soluble solids content [25, 37]. A total of thirty (30) pediatric liquid medicines were selected from community pharmacies and licensed retail pharmaceutical outlets in Ghana. Product selection was based on availability, frequency of use in pediatric practice, and representation across major therapeutic classes commonly prescribed for children. The sampled medicines were categorised into six therapeutic classes: analgesics, antitussives, antihistamines, antibiotics, non-steroidal anti-inflammatory drugs (NSAIDs), and antimalarials. Both locally manufactured and imported formulations were included to reflect the diversity of products accessible to caregivers in the Ghanaian pharmaceutical market. To ensure sample integrity, only products with intact packaging, valid expiry dates, and complete labelling information were included. Duplicate brands or products with identical formulations were excluded to minimise analytical redundancy. Prior to analysis, each product was assigned a unique sample identification code (ID). Detailed product information, including sample ID, origin, formulation type, and instructions for use, is provided in Supplementary Table S1**.** Hereafter, products are referred to by their assigned IDs.

### Sampling strategy justification in LMIC pharmaceutical markets

The sampling approach was designed to reflect real-world exposure patterns in low- and middle-income country (LMIC) pharmaceutical markets, where pediatric medicines are commonly obtained through community pharmacies and over-the-counter chemical sellers [38]. In such settings, access to pediatric medicines often occurs outside hospital environments, and product availability may vary across outlets depending on supply chains and regulatory oversight. Consequently, selecting medicines directly from retail outlets allows the study to capture formulations most likely to be used by caregivers and children in routine practice [39, 40]. This pragmatic sampling strategy has been recommended in pharmaceutical quality and formulation studies conducted in LMIC contexts because it provides representative evidence of products circulating within the medicine supply chain and reflects real-world medicine availability and utilisation patterns [40].

### Composite cariogenic–erosive risk scoring model

**Fig. 1:**
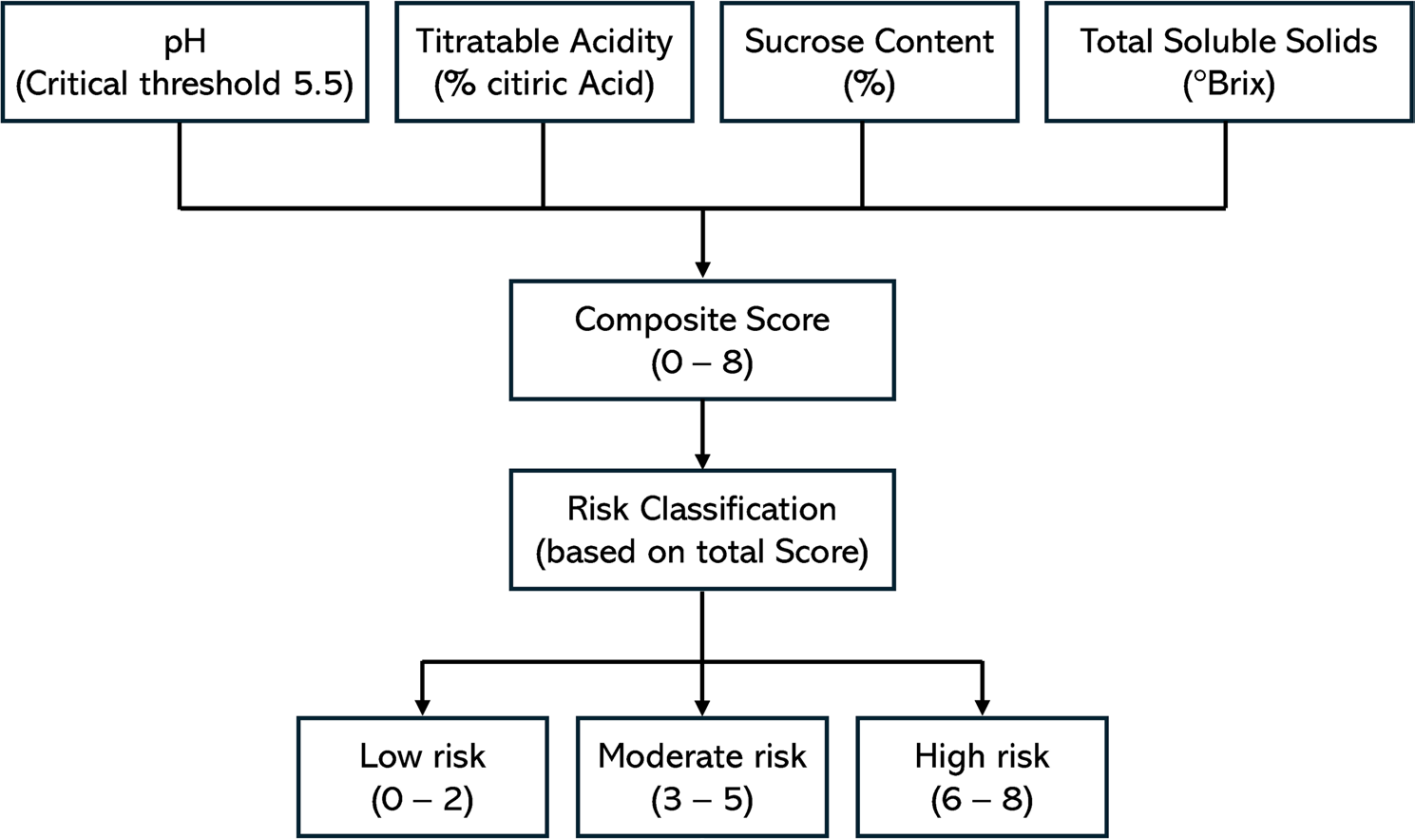
Schematic representation of the composite cariogenic–erosive risk scoring model used to classify paediatric liquid medicines. Four physicochemical parameters: pH, titratable acidity (TTA), sucrose content, and total soluble solids content (TSSC) were assigned numerical scores based on predefined thresholds. The summed score produced a composite risk value used to classify formulations into low-, moderate-, or high-risk categories for potential dental caries and enamel erosion

To enable an integrated evaluation of key physicochemical determinants of dental risk, a composite cariogenic–erosive risk scoring system was developed. The model is based on established evidence identifying pH, titratable acidity (TTA), sucrose content, and total soluble solids content (TSSC) as critical contributors to enamel demineralisation, erosive potential, and cariogenicity [27–31, 41, 42]. Numerical scores were assigned to each parameter using threshold values derived from previous studies and recognised critical limits (e.g., pH < 5.5 for enamel dissolution). Individual parameter scores were summed to generate a composite risk score, allowing classification of each product into low-, moderate-, or high-risk categories for potential dental caries and enamel erosion (Fig. 1). Although this composite model has not been externally validated, it provides a pragmatic, evidence-informed framework for integrating multiple risk factors and facilitating comparative assessment across formulations. Similar multi-parameter approaches have been advocated in the literature to better reflect the multifactorial nature of dental erosion and caries risk.

### Justification for parameter thresholds

The thresholds used in the scoring model were selected based on established experimental and clinical evidence regarding enamel demineralisation and sugar-associated cariogenicity [37, 43]. A pH threshold of 5.5 was adopted as the critical value below which hydroxyapatite dissolution increases significantly, representing the widely accepted critical pH for enamel demineralisation [5, 27, 43]. Medicines with a pH ≤ 4.0 were assigned a higher score because strongly acidic solutions accelerate enamel softening and mineral loss [27]. Titratable acidity thresholds were incorporated to reflect the buffering capacity of the formulation, which determines the total acid challenge and resistance to salivary neutralisation. Previous studies have demonstrated that solutions with higher titratable acidity can sustain erosive conditions for longer periods, even when the initial pH is similar [9, 44]. A sucrose threshold of 10% (w/w) was used as an indicator of increased cariogenic potential. Fermentable sugars provide substrates for oral microorganisms, leading to acid production and progressive enamel demineralisation, particularly under repeated exposure conditions [24, 37, 45]. Finally, total soluble solids content (TSSC), expressed in °Brix, was included as a surrogate indicator of formulation viscosity and sugar concentration. High-Brix solutions tend to exhibit greater oral retention and slower salivary clearance, thereby prolonging the contact time between acidic or sugary formulations and the tooth surface [46]. The resulting composite risk model was designed to be transparent, reproducible, and clinically interpretable, enabling simultaneous evaluation of erosive and cariogenic determinants within a single structured framework. This approach facilitates meaningful comparison of pediatric medicines across therapeutic classes and provides an evidence-based basis for identifying formulations with elevated oral health risks.

### Physicochemical analyses

All reagents used were of analytical grade. Measurements were performed at room temperature (25 ± 1 °C) and conducted in triplicate to ensure reproducibility. Mean values were calculated for analysis.

### pH Determination

The pH of each pediatric liquid medicine was measured using a calibrated digital pH meter (Hanna Instruments, USA) equipped with a glass electrode. Before measurement, the pH meter was calibrated using standard buffer solutions at pH 4.00, 7.00, and 10.00 in accordance with manufacturer specifications and standard analytical protocols [47]. Approximately 10 mL of each sample was transferred into a clean beaker, and the electrode was immersed directly into the solution. The pH was recorded once a stable reading was obtained. The relevance of pH measurement is based on the established critical pH threshold for enamel demineralisation (≈ 5.5), below which hydroxyapatite dissolution accelerates [5, 27].

### Total titratable acidity (TTA)

Total titratable acidity was determined by acid–base titration following established methods used in erosive potential studies [9, 28]. An aliquot (10 mL) of each sample was diluted to 100 mL with distilled water to prepare 10% solution. A 25mL aliquot was titrated with 0.01 N sodium hydroxide (NaOH) under constant stirring until the endpoint pH of 8.2–8.4 was reached. The endpoint was selected to approximate neutralisation of organic acids present in the formulation. TTA was expressed as percentage citric acid equivalents using the formula:

$$\mathrm{TTA} \left( \% \text{citric acid} \right) = \frac{V \times N \times F \times m_{eq-g} \left( \text{citric acid} \right) \times 100 } {V_s}$$

Where:

- *V* = volume (mL) of NaOH used.
- *N* = normality of NaOH.
- *F =* Normality correction factor.
- *m*_*eq-g*_ = milliequivalent weight of citric acid ≈ 0.064 g/m-eq; for calculation commonly simplified using standard citric acid equivalence factor)
- *V*_*s*_ = volume of sample (mL).

Titratable acidity is considered a more clinically relevant indicator of erosive potential than pH alone because it reflects buffering capacity and resistance to salivary neutralisation [9, 46].

### Sucrose content determination (Modified Lane–Eynon method)

Sucrose content was determined using a modified Lane–Eynon titrimetric method following acid inversion, as previously described for sugar analysis in pharmaceutical syrups and food matrices [48]. The Lane–Eynon method remains widely accepted for quantifying reducing sugars in syrups and pharmaceutical formulations [48]. The method quantifies reducing sugars before and after hydrolysis of sucrose to its monosaccharide components. A 5.0 g aliquot of each pediatric liquid medicine was accurately weighed and transferred into a 100 mL volumetric flask. The volume was made up to the mark with distilled water to obtain a homogeneous stock solution. Two independent stock solutions were prepared for each sample to enable the determination of reducing sugars before and after hydrolysis.

#### Determination of reducing sugars before hydrolysis (Glucose_a_)

One stock solution was transferred into a burette and titrated against a freshly prepared mixture of 10 mL Fehling’s solution (equal volumes of Fehling’s A and Fehling’s B) placed in a conical flask. The mixture was heated under gentle boiling while continuously agitated. The endpoint of the reaction was identified by the disappearance of the blue coloration of Cu^2+^ ions and formation of a characteristic brick-red precipitate of cuprous oxide (Cu_2_O), indicating reduction of copper (II) ions by reducing sugars.

The percentage of reducing sugars (Glucose_a_) was calculated using:

$$\mathrm{Glucose}_a \left( \% \right) = \frac{3.905} {2} \times \frac{\mathrm{V}^{-1.0251}} {\mathrm{P}} \times 100$$  

Where:

- * V*= volume (mL) of sample required for titration.
- * P*= weight (g) of sample in the stock solution.

If no colour change occurred, the free reducing sugar content was considered negligible.

#### Determination of reducing sugars after acid hydrolysis (Glucose_b_)

**Fig. 2:**
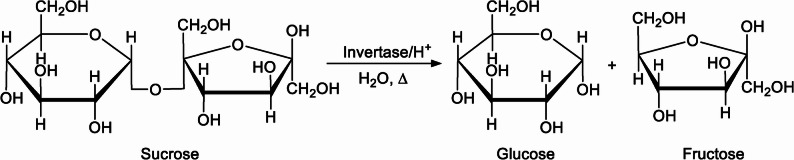
Acid hydrolysis of non-reducing Sucrose to obtain reducing Glucose

The second stock solution was subjected to acid inversion to hydrolyse sucrose (a non-reducing disaccharide) into glucose and fructose (reducing monosaccharides). One millilitre of concentrated hydrochloric acid (HCl) was added to the stock solution, and the mixture was heated in a boiling water bath for 40 min. The hydrolysis reaction, which converts non-reducing sucrose into reducing glucose, is illustrated in Fig. 2.

After cooling to room temperature, the hydrolysed solution was neutralised with sodium hydroxide (NaOH) solution. The neutralised solution was then titrated against Fehling’s solution under identical conditions as described for Glucose_*a*_. The percentage of total reducing sugars after inversion (Glucose_*b*_) was calculated using the same formula.

#### Calculation of sucrose content

Sucrose concentration was calculated from the difference between total reducing sugars after hydrolysis (Glucose_*b*_) and free reducing sugars before hydrolysis (Glucose_*a*_):

$$\mathrm{Sucrose} \left( \% w/w \right) = \mathrm{Glucose}_b - \mathrm{Glucose}_a$$

High sucrose concentrations are associated with increased cariogenic potential due to bacterial fermentation and acid production in dental biofilm [24, 37].

### Total soluble solids content (TSSC)

Total soluble solids content was measured using an Abbe refractometer calibrated with distilled water (0°Brix) before use. A drop of each sample was placed on the prism surface, and readings were recorded in degrees Brix (°Brix), representing grams of soluble solids per 100 g of solution [49]. TSSC serves as an indirect indicator of viscosity and sugar concentration. Increased viscosity may prolong retention time of fermentable carbohydrates on tooth surfaces, thereby increasing cariogenic risk [46].

### Quality control and reproducibility

All physicochemical measurements were performed in triplicate. Instruments were recalibrated after every ten samples to ensure analytical consistency, and the coefficient of variation for repeated measurements was maintained below 5%.

### Data analysis and statistical methods

Data were expressed as mean ± standard deviation (SD). Descriptive statistics were used to summarise the distribution of pH, total titratable acidity, sucrose content, and total soluble solids (°Brix) across the sampled pediatric liquid medicines and therapeutic classes. Data processing and preliminary analyses were performed using Microsoft Excel, while custom Python scripts were used to automate calculations for parameters such as titratable acidity and sucrose content. Comparative assessments were conducted between locally manufactured and imported products. A composite cariogenic–erosive risk score was calculated by summing the individual parameter scores derived from pH, titratable acidity, sucrose content, and total soluble solids. Formulations were subsequently classified into low-, moderate-, or high-risk categories, and their distribution across therapeutic classes was summarised using frequencies and percentages. Comparisons of physicochemical parameters among therapeutic classes were performed using one-way analysis of variance (ANOVA) in OriginPro (Version 2023b, OriginLab Corporation, Northampton, MA, USA) [50]. Where significant differences were observed, Tukey’s post hoc multiple comparison test was applied. A *p*-value < 0.05 was considered statistically significant.

## Results

### Distribution of pediatric liquid medicines

A total of 30 pediatric liquid oral medicines representing six therapeutic classes: analgesics, antitussives, antihistamines, non-steroidal anti-inflammatory drugs (NSAIDs), antimalarials, and antibiotics were analysed. Each therapeutic class comprised five products. The medicines included both locally manufactured (*n* = 18; 60%) and imported formulations (*n* = 12; 40%), reflecting the diversity of products available in the Ghanaian pharmaceutical market. Across the sampled medicines, commonly represented generic active ingredients included paracetamol (analgesics), dextromethorphan-containing combinations (antitussives), cetirizine and chlorphenamine (antihistamines), ibuprofen (NSAIDs), artemether–lumefantrine (antimalarials), and amoxicillin/clavulanic acid or metronidazole (antibiotics) (Table 1). Detailed product-level information, including assigned sample IDs, therapeutic classification, generic composition, origin, dosage form, and labelled instructions for use, is presented in Supplementary Table S1.

**Table 1:** Distribution of sampled pediatric liquid medicines by therapeutic class and origin

Therapeutic Class	Generic Drug(s)	Local (*n*)	Imported (*n*)	Total (*n*)
Analgesics	Paracetamol	3	2	5
Antitussives	Combination products	3	2	5
Antihistamines	Chlorphenamine	3	2	5
NSAIDs	Ibuprofen	3	2	5
Antimalarials	Artemether-Lumefantrine	3	2	5
Antibiotics	Amoxicillin-Clavulanic Acid, Metronidazole, Flucloxacillin	3	2	5
Total	—	18	12	30

### Physicochemical characteristics of pediatric liquid medicines

**Fig. 3:**
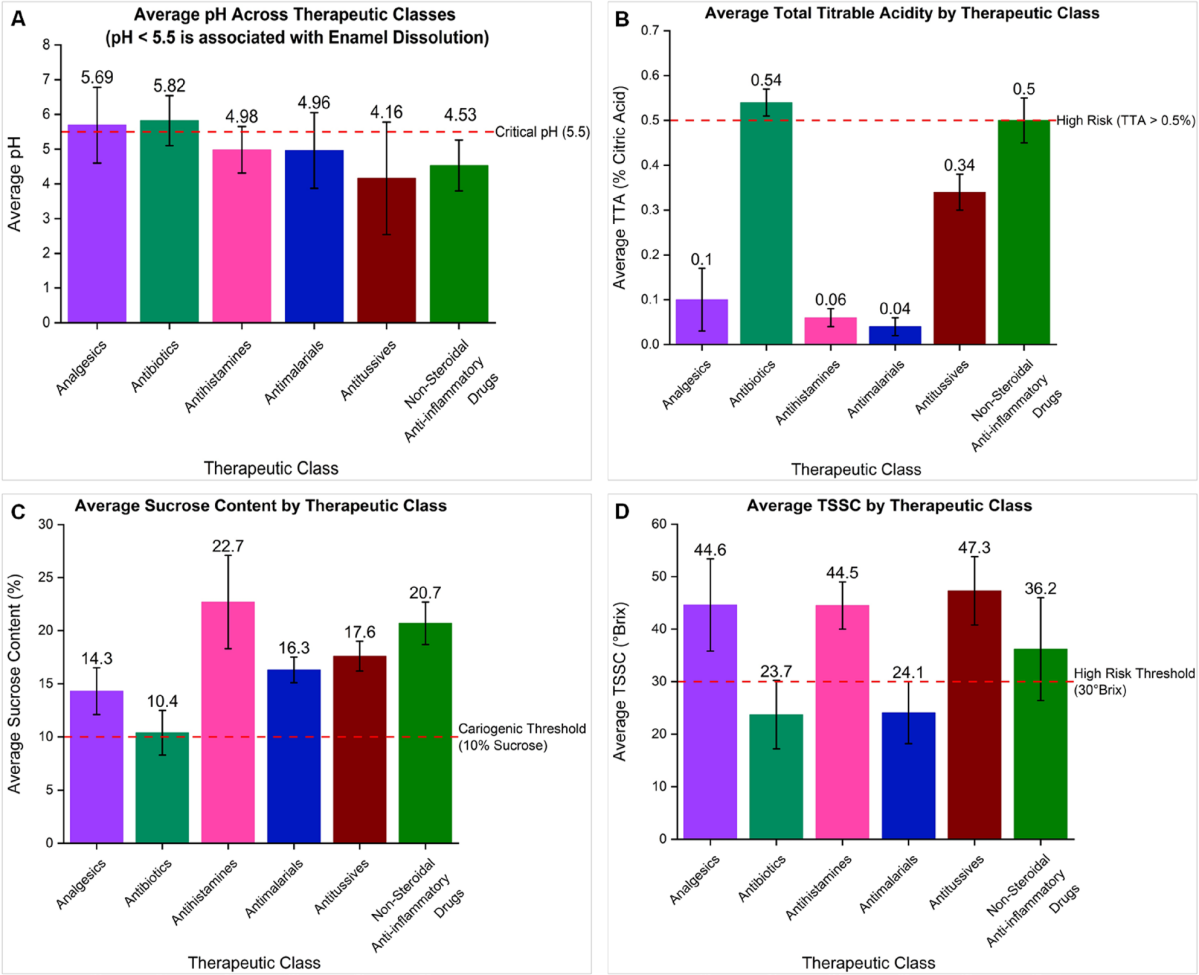
Physicochemical characteristics of pediatric oral liquid medications across therapeutic classes. (**A**) Average pH values of formulations within each therapeutic class, with the dashed horizontal line indicating the critical pH threshold for enamel dissolution (pH 5.5). (**B**) Mean total titratable acidity (TTA) expressed as percentage citric acid equivalent, with the dashed line representing the high-risk threshold (> 0.5% acid). (**C**) Average sucrose content (%) of the formulations, with the dashed line indicating the cariogenic threshold (10% sucrose). (**D**) Average total soluble solids content (TSSC, °Brix) across therapeutic classes, with the dashed line indicating the high-risk threshold (30 °Brix). Bars represent mean values for each therapeutic class, and error bars indicate variability among the analysed formulations

Table 2 summarizes the physicochemical characteristics of the evaluated pediatric oral liquid medications, including pH, titratable acidity (TTA), sucrose content, and total soluble solids content (TSSC). Considerable variation was observed among the individual formulations. The pH values of the analysed medicines ranged from 2.76 to 7.57, with several products exhibiting acidic pH levels below the critical enamel demineralization threshold of 5.5. Total titratable acidity (TTA), expressed as percentage citric acid equivalents, also varied widely across formulations, ranging from 0.01% to 1.66%, with some antibiotic suspensions demonstrating relatively high acidity values. Sucrose levels differed substantially among products, ranging from 0.0% to 75.6%, with several formulations exceeding the 10% threshold associated with cariogenic potential. Similarly, total soluble solids content (TSSC) values varied markedly, with some cough syrups and antihistamine formulations showing particularly high levels exceeding 30°Brix, indicative of a high concentration of soluble solids. TSSC values ranged from 4.2°Brix to 61.8°Brix. Overall, the data highlight significant variability in acidity and sugar-related physicochemical properties among commonly used pediatric liquid medicines. The physicochemical characteristics of pediatric oral liquid medications across therapeutic classes, including pH, total titratable acidity (TTA), sucrose content, and total soluble solids content (TSSC), are summarized in Fig. 3.

**Table 2:** Physicochemical characteristics of selected pediatric oral liquid medications, including pH, titratable acidity (TTA), sucrose content, and total soluble solids content (TSSC)

Sample ID	pH	TTA (%Citric Acid)	Sucrose Content (%)	TSSC (°Brix)
AN1	5.70	0.11	15.2	46.0
AN2	5.00	0.15	7.5	56.5
AN3	4.96	0.18	5.9	50.0
AN4	7.57	0.02	29.3	47.7
AN5	5.20	0.04	13.8	22.9
AT1	5.10	0.03	3.9	32.5
AT2	3.00	0.24	8.5	61.8
AT3	3.37	0.83	5.0	42.5
AT4	2.76	0.56	38.7	61.4
AT5	6.55	0.06	31.7	38.3
AH1	4.24	0.04	53.4	49.6
AH2	5.80	0.05	0.7	45.0
AH3	5.32	0.08	0.7	47.7
AH4	5.20	0.06	32.3	41.7
AH5	4.34	0.08	26.2	38.5
NS1	4.06	0.73	8.5	42.2
NS2	3.97	0.52	12.9	38.2
NS3	4.07	0.44	75.6	31.7
NS4	5.66	0.38	6.5	47.0
NS5	4.88	0.45	0.0	21.7
AM1	4.51	0.02	2.6	28.8
AM2	3.80	0.05	0.0	4.2
AM3	4.90	0.07	8.0	27.5
AM4	4.85	0.05	6.3	24.2
AM5	6.75	0.01	64.6	35.7
AB1	5.03	0.82	0.3	16.2
AB2	5.04	1.66	0.2	19.8
AB3	6.40	0.08	8.0	12.0
AB4	6.33	0.07	29.3	52.7
AB5	6.29	0.08	14.0	17.6

*Abbreviations*: *AN* analgesics, *AT *antitussives, *AH *antihistamines, *NS *non-steroidal anti-inflammatory drugs, *AM *antimalarials, *AB *antibiotics

#### pH of Pediatric Liquid Medicines

The average pH values of pediatric oral liquid medications across therapeutic classes are shown in Fig. 3A. Antibiotics and analgesics exhibited the highest mean pH values (5.82 and 5.69, respectively), while antitussives and non-steroidal anti-inflammatory drugs (NSAIDs) had the lowest mean pH values (4.16 and 4.53, respectively). Antimalarials and antihistamines showed intermediate mean pH values (4.96 and 4.98, respectively). Notably, the mean pH values of antihistamines, antimalarials, antitussives and NSAIDs were below the critical enamel dissolution threshold (pH 5.5), indicating a potential for enamel demineralization. Overall, 70% of the products exhibited pH values below the critical enamel dissolution threshold (pH 5.5).

#### Total titratable acidity

The average total titratable acidity (TTA) of pediatric oral liquid medications varied among therapeutic classes (Fig. 3B). Antibiotics (0.54% citric acid) exhibited the highest TTA value, exceeding the high-risk threshold of 0.5% citric acid. This was followed by non-steroidal anti-inflammatory drugs (0.50%), which were exactly on the borderline of the high-risk threshold. In contrast, analgesics (0.10%), antihistamines (0.06%), antimalarials (0.04%), and antitussives (0.34%) had lower TTA values below the risk threshold. The data indicate that only antibiotics and NSAIDs possess elevated titratable acidity associated with increased potential for dental erosion. Overall, 20% of medications exceeded the high-risk TTA threshold of 0.5% citric acid.

#### Sucrose content

**Fig. 4:**
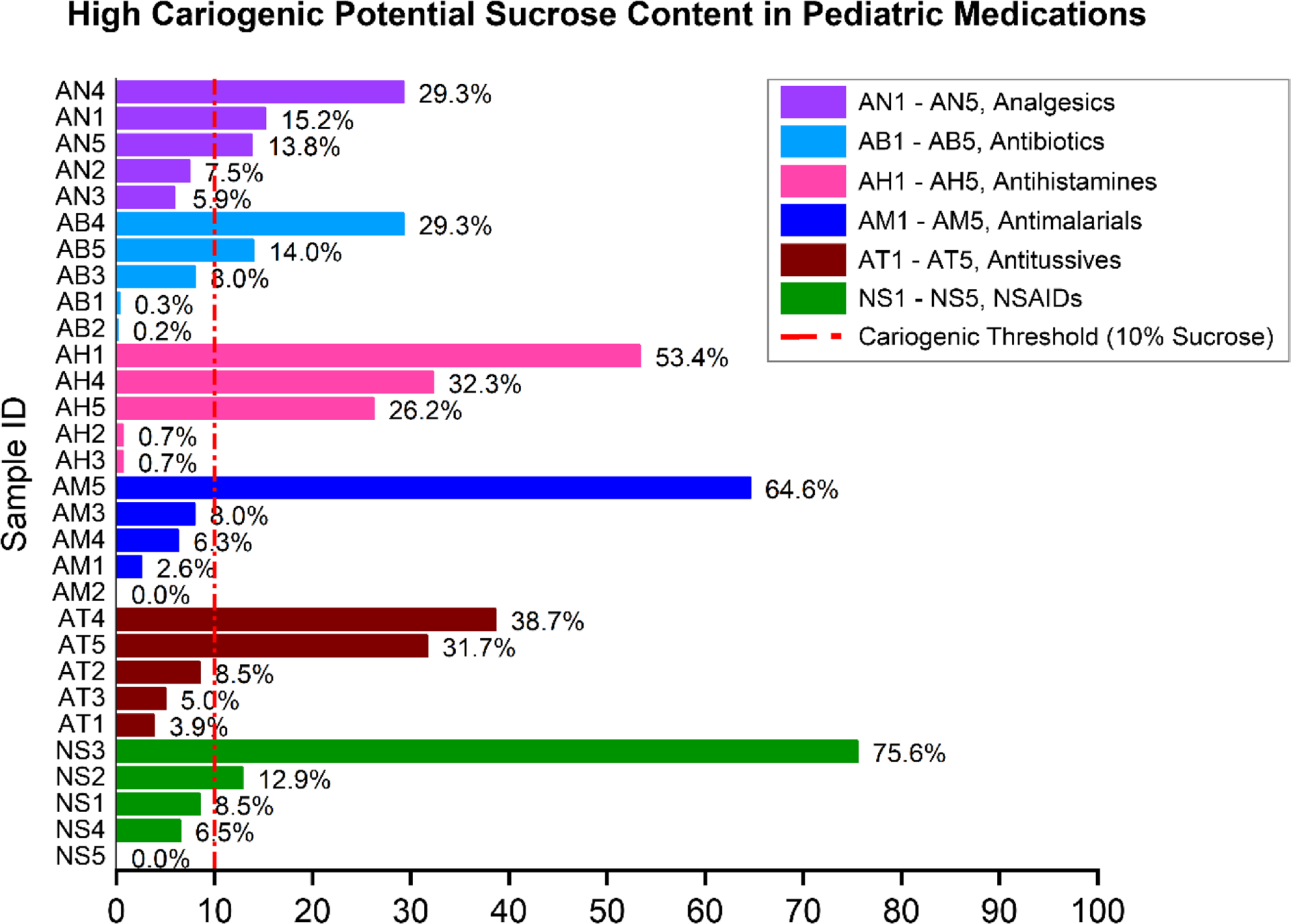
Sucrose content (%) of pediatric oral liquid medications by therapeutic class, with indication of the cariogenic threshold (10%)

The average sucrose content across therapeutic classes of pediatric oral liquid medications is presented in Fig. 3C. All evaluated classes exhibited mean sucrose content values above the cariogenic threshold of 10%, except for antibiotics, which showed a mean value close to this limit (10.4%). Antihistamines demonstrated the highest mean sucrose content (22.7%), followed by non-steroidal anti-inflammatory drugs (20.7%) and antitussives (17.6%). Antimalarials showed a moderate sucrose content (16.3%), whereas analgesics presented a slightly lower mean value (14.3%). Antibiotics displayed the lowest sucrose content (10.4%) among the therapeutic classes. Overall, 43.3% of all formulations contained sucrose levels exceeding the cariogenic threshold, indicating widespread use of sugar-based sweetening agents in pediatric liquid medications (Fig. 4).

#### Total soluble solids content

The average total soluble solids content (TSSC) varied across therapeutic classes of pediatric oral liquid medications (Fig. 3D). Antitussives, analgesics, and non-steroidal anti-inflammatory drugs (NSAIDs) exhibited high mean TSSC values (47.3°Brix, 44.6°Brix, 44.5°Brix, and 36.2°Brix, respectively), exceeding the high-risk threshold of 30°Brix, whereas antibiotics and antimalarials had comparatively lower values (23.7°Brix and 24.1°Brix, respectively), below the 30°Brix threshold.

### High-Risk medications

#### pH versus Total Titratable Acidity (TTA) for all pediatric medications

**Fig. 5:**
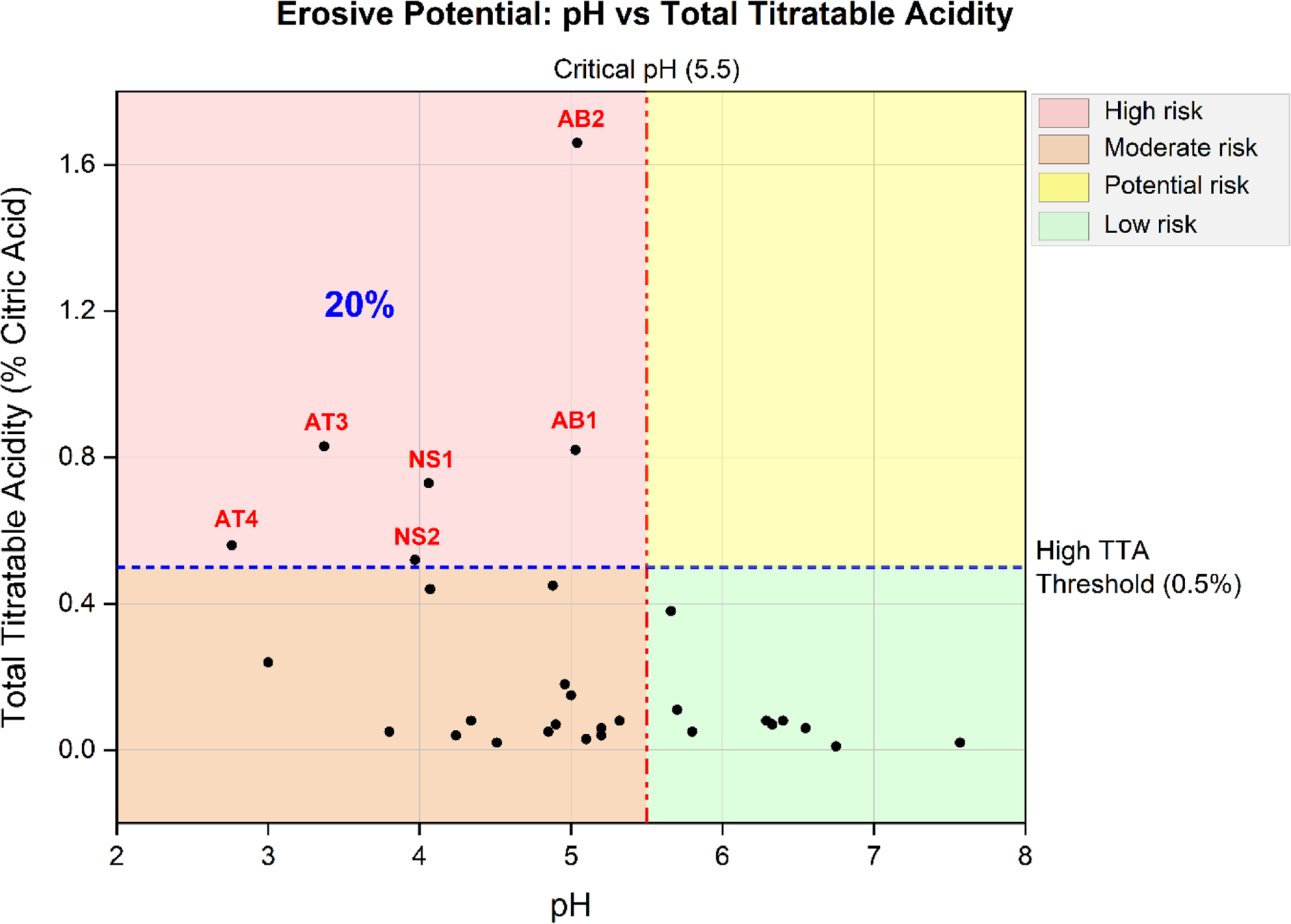
Scatter plot showing the relationship between pH and total titratable acidity of pediatric oral liquid medications. The vertical line represents the critical enamel dissolution pH (5.5), while the horizontal line represents the threshold for high titratable acidity (0.5%). Medicines located in the upper-left quadrant exhibit the highest erosive potential. The upper-left quadrant (pH < 5.5 and TTA > 0.5%) represents formulations with high-risk erosive potential. The upper-right quadrant (pH > 5.5 and TTA > 0.5%) represents formulations with erosive potential. The lower-left quadrant (pH < 5.5 and TTA < 0.5%) represents formulations with moderate-risk erosive potential. The lower-right quadrant (pH ≥ 5.5 and TTA ≤ 0.5% were classified as low risk and represents formulations with low-risk erosive potential

Figure 5 shows the joint relationship between pH and total titratable acidity (TTA) of the individual pediatric liquid medicines that were analysed and their corresponding erosive risk zones. The vertical dashed line indicates the critical pH threshold of 5.5, below which enamel demineralization may occur, while the horizontal dashed line represents the high titratable acidity threshold (0.5% citric acid equivalent). Approximately 20% of the evaluated medicines fell within this high-risk quadrant (pH < 5.5 and TTA > 0.5%), including AB2, AT3, AB1, NS1, and AT4, indicating both high acidity and buffering capacity. NS2 was positioned near the moderate-risk boundary. These formulations are therefore associated with increased erosive potential. In contrast, medicines with pH ≥ 5.5 and TTA ≤ 0.5% were classified as low risk, suggesting a lower erosive potential. These findings highlight the importance of considering both pH and titratable acidity when assessing the erosive potential of pediatric liquid medications [27, 51]. When pH and titratable acidity were considered jointly, several medicines exhibited combined low pH and high TTA, indicating increased erosive potential.

#### Total Soluble Solids Content (TSSC) versus Sucrose Content for all Pediatric Medications

**Fig. 6:**
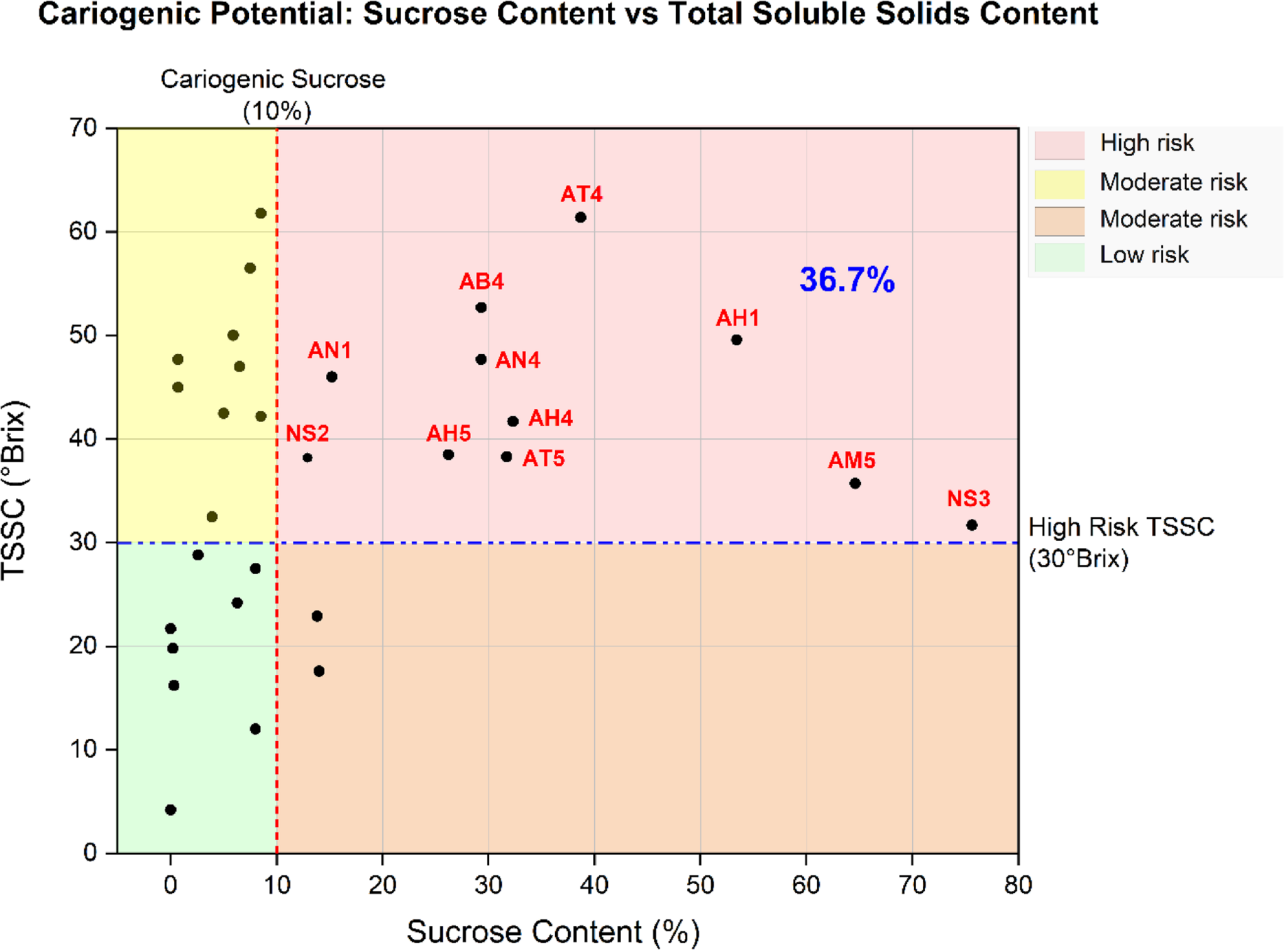
Scatter plot showing the relationship between Total Soluble Solids Content (TSSC) and Sucrose Concentration in Pediatric Oral Liquid Medications. The vertical and horizontal reference lines indicate the threshold values of 10% sucrose and 30 °Brix TSSC, respectively, dividing the plot into four quadrants. The upper-right quadrant (TSSC > 30 °Brix, sucrose > 10%) represents formulations with high-risk cariogenic potential. The upper-left quadrant (TSSC > 30 °Brix, sucrose ≤ 10%) represents formulations with moderate-risk cariogenic potential. The lower-right quadrant (TSSC ≤ 30 °Brix, sucrose > 10%) represents formulations with moderate-risk cariogenic potential. The lower-left quadrant (TSSC ≤ 30 °Brix, sucrose ≤ 10%) represents formulations with low-risk cariogenic potential

Also, formulations with high sucrose content and elevated TSSC demonstrated increased cariogenic potential. Figure 6 illustrates the relationship between sucrose content and total soluble solids content (TSSC, °Brix) of the analysed pediatric liquid medicines as indicators of cariogenic potential. The vertical dashed line represents the cariogenic sucrose threshold (10%), while the horizontal dashed line indicates the high-risk TSSC threshold (30°Brix). The high-risk quadrant (TSSC > 30 °Brix and sucrose > 10%) represents formulations with both high sugar concentration and high sucrose content, indicating greater cariogenic potential. Overall, 36.7% of the formulations were located in the high-risk quadrant, including AT4, AB4, AH1, AN4, AN1, AH4, AH5, AT5, AM5, NS2, and NS3. The upper-left quadrant (TSSC > 30 °Brix and sucrose ≤ 10%) includes formulations with high total soluble solids but relatively lower sucrose levels. The lower-right quadrant (TSSC ≤ 30 °Brix and sucrose > 10%) represents products with lower overall soluble solids but elevated sucrose content. The lower-left quadrant (TSSC ≤ 30 °Brix and sucrose ≤ 10%) corresponds to the low-risk group with comparatively lower sugar exposure. These findings highlight the significant sugar load present in many pediatric liquid formulations and their potential contribution to dental caries when used frequently or for prolonged periods [41, 52].

### Physicochemical characteristics of pediatric oral liquid medicines by therapeutic class and manufacturer origin

**Fig. 7:**
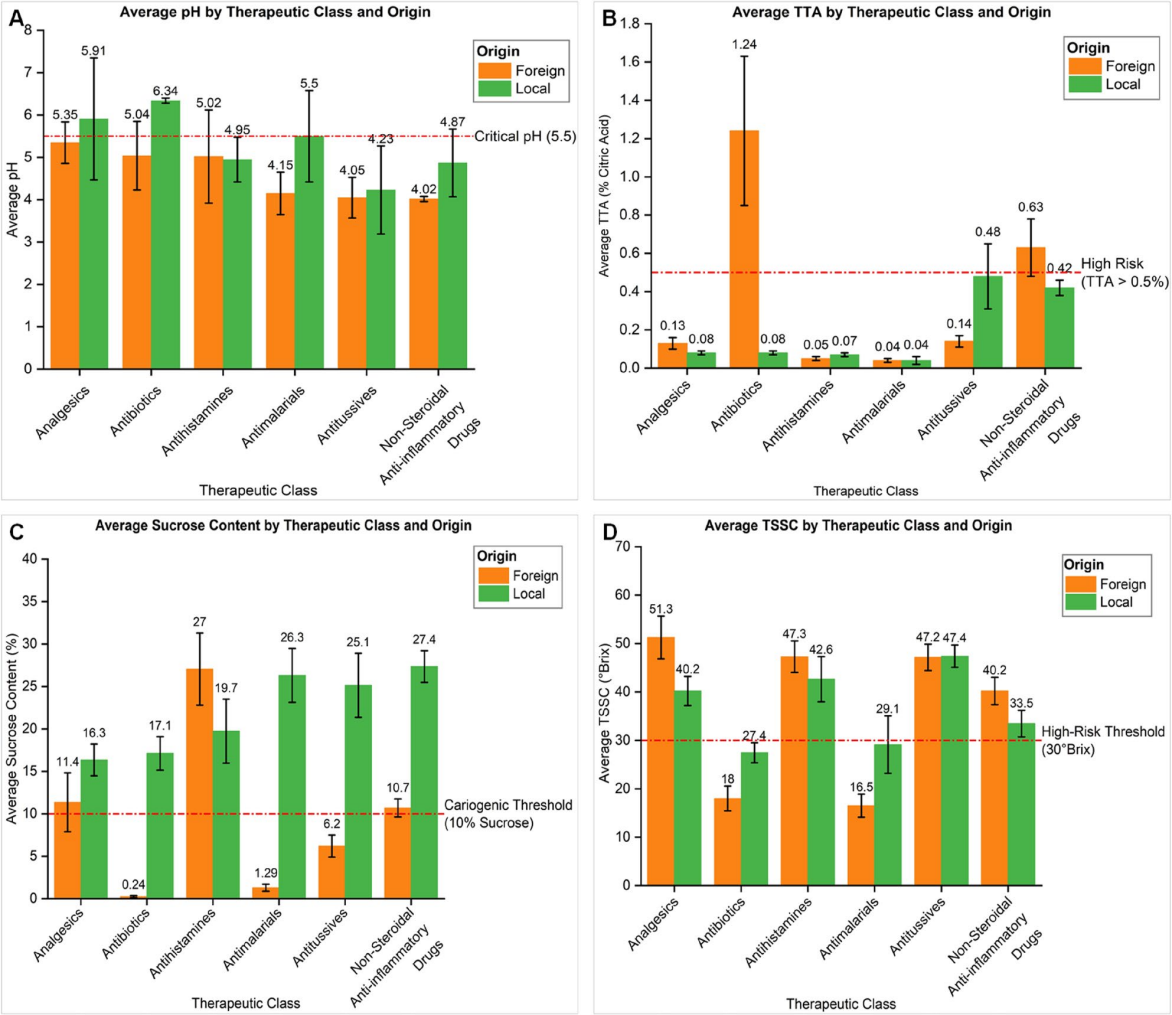
Presents the mean physicochemical characteristics of pediatric oral liquid medicines according to therapeutic class and manufacturer origin. Physicochemical Properties of Pediatric Oral Liquid Medicines by therapeutic class and manufacturer origin. (**A**) Mean pH values with the dashed line indicating the critical enamel demineralization threshold (pH 5.5). (**B**) Mean titratable acidity (TTA) expressed as percentage citric acid, with the dashed line indicating dashed line representing the high-risk threshold (0.5%). (C) Mean sucrose content (%) with the dashed line indicating the cariogenic threshold (10% sucrose). (**D**) Mean total soluble solid content (TSSC, °Brix) with the dashed line indicating the high-risk threshold (30 °Brix). Bars represent mean values for locally manufactured and imported formulations within each therapeutic class, and error bars indicate standard deviation

The average pH values varied across therapeutic classes, with several formulations exhibiting acidic pH levels below the critical enamel demineralization threshold of 5.5 (Fig. 7A). For the foreign-manufactured pediatric medicines, the average pH values of all therapeutic classes were below the critical pH of 5.5, with analgesics showing the highest average pH values of 5.35, while NSAIDs showed the lowest average pH value of 4.01. On the other hand, for the locally manufactured pediatric medicines, three therapeutic classes, namely antitussives, NSAIDs, and antihistamines, had average pH values of 4.23, 4.87, and 4.95, respectively, which were below the critical pH. Antimalarials had a borderline average pH of 5.50, whereas analgesics and antibiotics showed comparatively higher pH values (5.91 and 6.34, respectively). Overall, based on the data obtained, locally produced formulations tended to have slightly higher average pH values than imported products across most therapeutic classes.

In terms of titratable acidity, mean values differed substantially between therapeutic classes and manufacturers. Foreign-manufactured antibiotics demonstrated the highest overall mean titratable acidity (1.24%) while foreign NSAIDs exhibited relatively elevated titratable acidity (0.62% citric acid), both exceeding the high-risk threshold of 0.5%. In contrast, most locally manufactured formulations showed comparatively lower titratable acidity values, generally remaining below the high-risk threshold **(**Fig. 7B**)**.

Sucrose concentrations varied considerably across therapeutic classes. All locally manufactured medicines contained high mean sucrose levels exceeding the cariogenic threshold of 10%, including analgesics (16.3%), antibiotics (17.1%), antihistamines (19.7%), antitussives (25.1%), antimalarials (26.3%) and NSAIDs (27.4%). Among foreign products, antihistamines demonstrated the highest sucrose concentration (27.0%), whereas antibiotic and antimalarial formulations contained minimal sucrose (0.24% and 1.29%, respectively) **(**Fig. 7C**)**.

Similarly, total soluble solid content (TSSC) showed notable variation between therapeutic classes. Foreign-manufactured analgesics, antihistamines, antitussives, and NSAIDs exhibited particularly high TSSC values (51.3°, 47.3°, 47.2°, 40.2°Brix, respectively), all exceeding the high-risk threshold of 30°Brix. Locally manufactured products generally demonstrated lower but still substantial TSSC values in several classes, particularly antitussives (47.4°Brix) and antihistamines (42.6°Brix) **(**Fig. 7D**)**. Overall, the findings demonstrate considerable variation in acidity, buffering capacity, and sugar-related properties among pediatric liquid medicines, with several therapeutic classes exhibiting physicochemical characteristics associated with increased cariogenic and erosive potential.

### Composite cariogenic and erosive risk classification

**Fig. 8:**
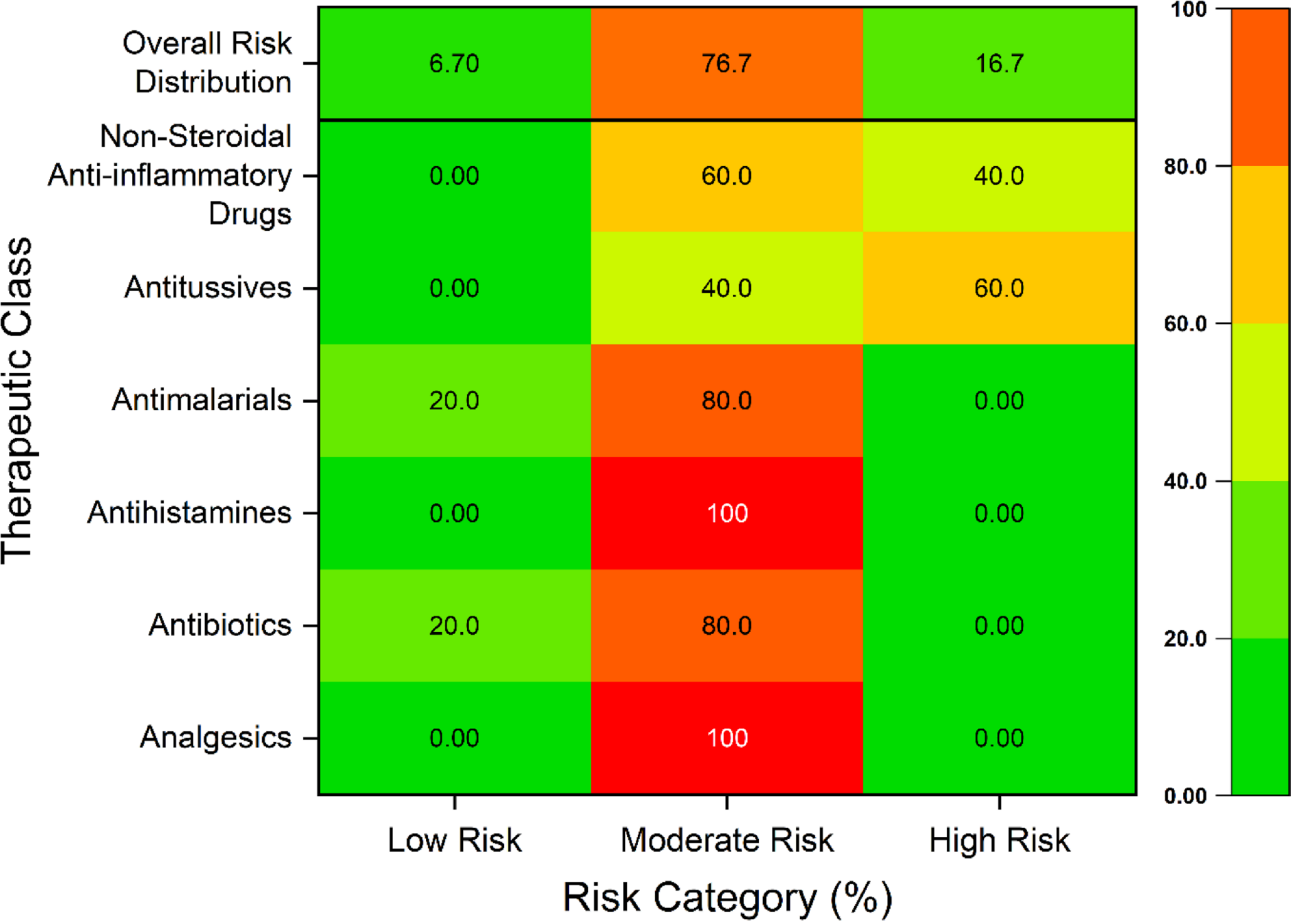
Heat map showing the percentage distribution of pediatric oral liquid medications across erosive risk categories by therapeutic class. Colour intensity represents the proportion of formulations within each risk category, with green indicating low risk and red indicating high risk

Using the composite cariogenic–erosive risk scoring scheme, individual medicines were classified as low, moderate, or high risk based on combined physicochemical parameters. The distribution of pediatric oral liquid medications across erosive risk categories according to therapeutic class is illustrated in Fig. 8. Overall, the majority of formulations were classified as moderate risk (76.7%), whereas high-risk medicines accounted for 16.7% and low-risk formulations represented only 6.7% of the total samples analysed. Within therapeutic classes, analgesics and antihistamines showed exclusively moderate-risk formulations (100%), while antibiotics and antimalarials each contained 80% moderate-risk and 20% low-risk formulations. In contrast, antitussives exhibited the highest proportion of high-risk formulations (60%), followed by non-steroidal anti-inflammatory drugs (NSAIDs), where 40% of the products were classified as high risk. Low-risk formulations were identified only among antibiotics and antimalarials, whereas no low-risk products were observed among analgesics, antihistamines, antitussives, or NSAIDs.

### Comparisons of physicochemical parameters among therapeutic classes

Comparisons of physicochemical parameters among therapeutic classes were performed using one-way analysis of variance (ANOVA). The analysis revealed no statistically significant differences in pH (*F* = 1.91, *p* = 0.130), total titratable acidity (*F* = 2.44, *p* = 0.063), or sucrose content (*F* = 0.22, *p* = 0.952) across therapeutic classes. However, a statistically significant difference was observed for total soluble solids content (TSSC) (*F* = 3.87, *p* = 0.010), indicating variability in overall soluble solid concentration among the therapeutic categories (Table 3).

**Table 3:** One-way ANOVA comparing physicochemical parameters among therapeutic classes of pediatric oral liquid medications

Parameter	Source of Variation	Sum of Squares	df	MS	F value	*p*-value
pH	Between Groups	10.37	5	2.07	1.91	0.130
	Within Groups	26.07	24	1.09		
	Total	36.44	29			
TTA	Between Groups	1.30	5	0.26	2.44	0.063
	Within Groups	2.54	24	0.11		
	Total	3.84	29			
Sucrose	Between Groups	488.47	5	97.69	0.22	0.952
	Within Groups	10821.97	24	450.92		
	Total	11310.43	29			
TSSC	Between Groups	2827.61	5	565.52	3.87	0.010*
	Within Groups	3503.62	24	145.98		
	Total	6331.23	29			

*Significant at *p* < 0.05

Following the significant ANOVA result for total soluble solids content (TSSC), Tukey’s HSD post-hoc test was conducted (Table 4). Pairwise comparisons revealed a statistically significant difference between antitussive and antibiotic formulations (*p* = 0.0498), with antitussive preparations exhibiting higher TSSC values. No other pairwise comparisons showed statistically significant differences among the therapeutic classes (*p* > 0.05).

**Table 4:** Tukey HSD post-hoc comparison matrix for total soluble solids content (TSSC) among therapeutic classes of pediatric oral liquid medications

Therapeutic Class	Analgesics	Antitussives	Antihistamines	NSAIDs	Antimalarials	Antibiotics
Analgesics	—	0.999	1.000	0.874	0.115	0.103
Antitussives		—	0.999	0.693	0.056	0.0498*
Antihistamines			—	0.880	0.118	0.106
NSAIDs				—	0.618	0.584
Antimalarials					—	1.000
Antibiotics						—

Values represent Tukey HSD *p*-values for pairwise comparisons of mean TSSC between therapeutic classes

*Significant difference at *p* < 0.05

**Fig. 9:**
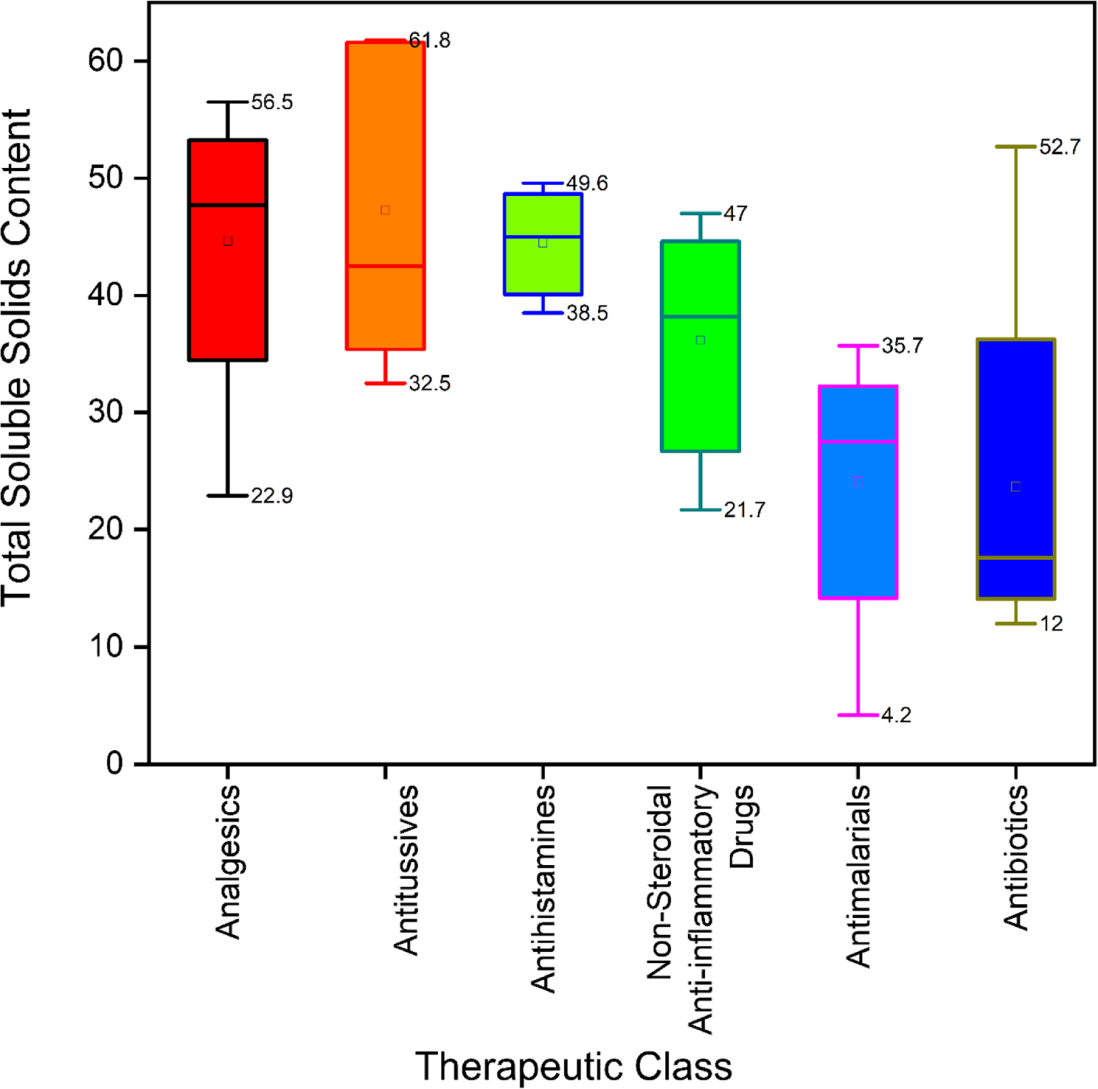
Box plot showing variation in total soluble solids content among therapeutic classes of pediatric oral liquid medications

The distribution of TSSC across therapeutic classes is presented in Fig. 9. The box plot highlights differences in both median values and variability among the drug categories. Antitussives exhibited the highest median TSSC values along with a wide interquartile range, indicating both high soluble solid content and considerable variability. Antihistamines also showed relatively high median values but with a more compact distribution. In contrast, NSAIDs demonstrated moderate median TSSC levels with less variability compared to antitussives. Antimalarials and antibiotics generally had lower median TSSC values; however, both classes displayed wide overall ranges, particularly antibiotics, indicating substantial variability in soluble solid content. These patterns suggest that while some therapeutic classes consistently exhibit higher TSSC, variability within classes remains an important consideration.

## Discussion

A substantial proportion of the pediatric liquid medicines analysed exhibited physicochemical characteristics associated with increased risk of dental caries and tooth erosion. Notably, 70% of the formulations had pH values below the critical enamel threshold of 5.5. Solutions with pH values below the critical threshold for enamel dissolution can initiate surface softening [9–12]. Therefore, the predominance of acidic pH values observed in this study highlights the potential for enamel demineralisation with repeated exposure. These findings are consistent with reports from other regions, including Brazil (approximately 65–75%) [28, 53], as well as studies conducted in India and the United Kingdom (approximately 60–80%) [30, 31, 54]. Similar trends have been documented in Saudi Arabia, where all evaluated pediatric liquid medications were acidic (pH 4.22–6.10) and contained sucrose, and in Egypt, where syrups exhibited pH values ranging from 3.47 to 6.92 alongside measurable sugar content capable of inducing enamel surface alterations [55, 56]. Additional evidence from Brazil further confirms that pediatric oral liquid medicines commonly combine low pH with high sucrose concentrations, both of which are key contributors to dental erosion and caries development [57]. Collectively, the consistency of these findings across geographically diverse settings suggests that the acidic nature of pediatric syrup formulations reflects global pharmaceutical practices rather than region-specific trends. The particularly low pH values observed among antitussives and non-steroidal anti-inflammatory drugs (NSAIDs) in this study may be attributed to the inclusion of acidulants such as citric acid or benzoic acid, which enhance stability, microbial preservation, and palatability [27].

While low pH alone can initiate enamel demineralisation, it does not fully determine erosive potential. Total titratable acidity (TTA) is a critical parameter, as it reflects both the total acid content and buffering capacity of a formulation, thereby influencing the duration and persistence of the acid challenge in the oral cavity. Higher TTA prolongs acid exposure and delays neutralisation by saliva, thereby increasing erosive risk [9]. In this study, 20% of formulations exceeded the high-risk TTA threshold (≥ 0.5%), with antibiotics and NSAIDs showing the highest values. These findings are consistent with previous studies indicating that TTA is a stronger predictor of erosive potential than pH alone, as it determines resistance to salivary neutralisation [27, 42, 58]. The identification of formulations with both low pH and high TTA further emphasizes the importance of combined physicochemical assessment when evaluating erosive risk.

The clinical implications of these findings are further amplified by patterns of medication use in pediatric populations. Many childhood conditions require repeated or prolonged administration of liquid medicines, increasing the frequency of exposure to acidic formulations [59]. Recurrent intake, particularly in the absence of post-administration oral hygiene practices such as rinsing, may prolong acid contact with tooth surfaces and accelerate enamel demineralisation. This risk is further heightened when medications are administered at bedtime, a period characterized by reduced salivary flow and diminished buffering capacity, which facilitates prolonged retention of acidic residues on tooth surfaces [60]. The identification of commonly used formulations, including certain antibiotics, NSAIDs, and antitussives with high erosive potential, raises important concerns regarding their cumulative impact on pediatric oral health.

Of particular concern is the observation that several high-risk formulations identified in this study are either commonly prescribed or widely available over-the-counter. Notably, some antibiotic suspensions and frequently used NSAIDs exhibited physicochemical properties linked to increased erosive risk. Similarly, certain antitussive preparations often administered in the evening or at bedtime may further heighten this risk by prolonging contactwith dental tissues. Given the widespread use of these medications in Ghana, these findings raise important public health concerns about their cumulative impact on pediatric oral health.

In addition to acidity, sucrose content and total soluble solids content emerged as key determinants of the cariogenic potential. A considerable proportion (43.3%) of the analysed medicines exceeded the 10% sucrose threshold associated with increased risk of dental caries. Fermentable sugars serve as substrates for oral bacteria, leading to acid production and progressive enamel demineralisation [24, 37, 45]. The individual medications that crossed the cariogenic threshold include frequently dispensed over-the-counter medications as well as pediatric medications used in chronic conditions such as asthma. This indicates that children in Ghana are being frequently exposed to medications that are cariogenic, and in some situations, there is prolonged exposure to these cariogenic medications. This finding is consistent with reports from other regions, including the United Kingdom and Brazil, where 50–70% of pediatric medicines contain fermentable sugars [31, 57]. The widespread use of sucrose in these formulations is largely attributed to its effectiveness in masking the bitter taste of active pharmaceutical ingredients, thereby improving palatability and adherence, particularly among young children. High total soluble solids content, which reflects both sugar concentration and formulation viscosity, plays a significant role in the cariogenic potential of paediatric liquid medicines. In this study, several products, particularly antitussives and antihistamines, exhibited TSSC values exceeding 30°Brix, indicating a high concentration of dissolved solids. Elevated TSSC is associated with increased viscosity and prolonged oral retention, which enhances adherence to tooth surfaces, reduces salivary clearance, and promotes bacterial fermentation and acid production [61]. The combined presence of high sucrose content and elevated TSSC, observed in over one-third of the formulations, suggests a heightened risk of sustained biofilm acidogenicity and enamel demineralisation. This risk may be further exacerbated in real-world settings where paediatric medicines are frequently administered at night or without subsequent oral hygiene measures, prolonging exposure of dental tissues to fermentable substrates. Overall, these findings underscore the importance of evaluating both sucrose concentration and total soluble solids content when assessing the cariogenic potential of paediatric formulations, as their combined effects provide a more comprehensive and clinically relevant measure of risk.

The cariogenicity of pediatric liquid medicines is further exacerbated by behavioural and clinical factors. Frequent administration, particularly in children with chronic conditions requiring long-term medication use, increases cumulative sugar exposure and has been associated with a higher prevalence of dental caries and early childhood caries (ECC) [62, 63]. Moreover, the palatable nature of these formulations may encourage unsupervised consumption, further increasing exposure frequency. Oral hygiene practices and timing of administration also play a critical role. In many cases, medications are administered at bedtime, when salivary flow is physiologically reduced, limiting the natural buffering and clearance of sugars from the oral cavity [60]. When combined with inadequate oral hygiene practices, this results in prolonged retention of fermentable carbohydrates, facilitating bacterial metabolism and the production of organic acids that drive the caries process [60]. Consequently, the interaction between high sugar content, elevated TSSC, frequent exposure, and reduced salivary clearance creates a highly favourable environment for dental caries development.

Although most physicochemical parameters did not differ significantly across therapeutic classes, TSSC showed a statistically significant variation (*p* = 0.010), with antitussives exhibiting higher values compared to antibiotics. This likely reflects formulation practices, as cough syrups often require higher concentrations of sweeteners and viscosity modifiers to enhance palatability and therapeutic effect [64]. In contrast, the absence of significant differences in pH, TTA, and sucrose content suggests that these risk-related properties are widespread across all therapeutic classes rather than confined to specific drug classes.

The integration of multiple physicochemical parameters provides a more clinically relevant assessment of oral health risk. In this study, high-risk medicines accounted for 16.7%, while low-risk formulations represented only 6.7%, with the majority (76.7%) classified as moderate risk. This distribution suggests that cumulative exposure over time, rather than single-use effects, is the primary driver of dental damage in pediatric populations — an observation supported by evidence linking repeated exposure to acidic and sugar-containing medicines with increased enamel demineralisation and caries development [19, 65, 66]. These findings highlight the importance of considering both individual parameters and their combined effects when evaluating overall dental risk. Formulations that simultaneously exhibit low pH, high titratable acidity, and elevated sucrose content represent a compounded risk scenario, promoting both immediate enamel softening and sustained cariogenic activity. Therapeutic class patterns further showed that antitussives and NSAIDs exhibited the highest combined risk profiles, whereas antibiotics and antimalarials generally demonstrated lower risk, albeit with some exceptions. Similar trends have been reported internationally, where cough syrups and analgesic formulations often contain higher concentrations of sugars and acids, increasing their cariogenic and erosive potential [19, 28]. Importantly, recent literature emphasises that the combined assessment of pH, titratable acidity, and sugar-related parameters is essential for accurately predicting oral health risk. A growing body of evidence indicates that formulations with low pH and high buffering capacity, particularly when combined with fermentable sugars, exert a synergistic effect on enamel dissolution and biofilm acidogenicity [10, 27, 67]. Collectively, these findings reinforce the need for multidimensional risk assessment approaches when evaluating pediatric oral liquid medications.

Differences between locally manufactured and imported products were also observed, although these variations were not uniform across all parameters. Locally produced formulations generally exhibited slightly higher pH values but consistently high sucrose levels, while imported products showed higher TTA in some classes (e.g., antibiotics and NSAIDs). Such differences may reflect heterogeneity in formulation strategies, excipient selection, and manufacturing standards. While both local and imported medicines contributed to the observed risk profiles, the findings underscore the need for consistent quality benchmarks that incorporate oral health considerations alongside therapeutic efficacy and safety, as increasingly recognised in international regulatory guidance on excipient use in pediatric formulations [68–70]. Previous studies have shown that countries with stricter regulatory policies promote sugar-free formulations, reducing cariogenic risk [71]. The absence of such widespread regulatory enforcement in many low- and middle-income countries may explain the persistence of high-sugar formulations.

### Clinical and Public Health Implications

The public health implications of these findings are significant, particularly in low- and middle-income settings where access to preventive dental care may be limited and the use of liquid pediatric formulations is widespread. Children with chronic conditions requiring repeated or long-term medication use may be at increased risk of cumulative enamel damage. These results support the need for regulatory policies that encourage the use of sugar-free or low-acidity formulations, improved labelling of excipient content, and greater awareness among healthcare providers regarding the potential oral health effects of prescribed medicines. Integrating oral health counselling into pediatric care, such as advising rinsing after medication use or avoiding night-time dosing without brushing, may help mitigate these risks.

### Limitations

The study was limited to physicochemical properties and did not include in vivo assessment of enamel changes or clinical outcomes such as caries incidence. Additionally, behavioural factors such as diet, oral hygiene practices, and frequency of medication use were not directly assessed, although they are known to influence caries risk [5, 24]. The sample size, while representative of commonly available products, may not capture the full diversity of formulations present in the Ghanaian market. Despite these limitations, the findings provide robust baseline data and a structured framework for evaluating the oral health implications of pediatric liquid medicines in resource-limited settings.

## Conclusions

Overall, pediatric oral liquid medications marketed in Ghana exhibit physicochemical characteristics associated with both dental caries and enamel erosion. The consistency of these findings with global evidence underscores that this is a widespread public health concern. There is a clear need for coordinated efforts involving regulators, manufacturers, healthcare providers, and caregivers to promote safer formulations and reduce medication-related oral health risks in children.

## Supplementary Information

Supplementary Material 1.

## Data Availability

Data generated in this study are available from the corresponding author upon reasonable request.

## Abbreviations

NSAIDs: Non-steroidal anti-inflammatory drugs
TTA: Total titratable acidity
TSSC: Total soluble solids content

## References

[1] Kassebaum NJ, Smith AGC, Bernabé E, Fleming TD, Reynolds AE, Vos T, Murray CJL, Marcenes W, null n, Abyu GY, et al. Global, regional, and national prevalence, incidence, and disability-adjusted life years for oral conditions for 195 countries, 1990–2015: a systematic analysis for the global burden of diseases, injuries, and risk factors. J Dent Res. 2017;96(4):380–7. 10.1177/0022034517693566. 10.1177/0022034517693566 PMC5912207 28792274

[2] Mathur MR, Tsakos G, Parmar P, Millett CJ, Watt RG. Socioeconomic inequalities and determinants of oral hygiene status among Urban Indian adolescents. Commun Dent Oral Epidemiol. 2016;44(3):248–54. 10.1111/cdoe.12212. 10.1111/cdoe.12212 26762656

[3] Lussi A, Carvalho TS. Erosive tooth wear: a multifactorial condition of growing concern and increasing knowledge. Monogr Oral Sci. 2014;25:1–15. 10.1159/000360380. 10.1159/000360380 24993253

[4] Stephan R. Intraoral hydrogen ion concentration on tooth surfaces and in carious lesion. J Am Dent Assoc. 1944;23:257–66. 10.1177/00220345440230040401.

[5] Fejerskov O, Nyvad B, Kidd E. Dental caries: the disease and its clinical management. Wiley; 2015.

[6] Bernimoulin J-P. Recent concepts in plaque formation. J Clin Periodontol. 2003;30(s5):7–9. 10.1034/j.1600-051X.30.s5.3.x. 12787195 10.1034/j.1600-051x.30.s5.3.x

[7] Ruiz DC, Marqués Martínez L, García Miralles E. Dental Erosion and Diet in Young Children and Adolescents: A Systematic Review. Appl Sci. 2023;13(6):3519. 10.3390/app13063519.

[8] American Dental Association (ADA). Dental erosion: key points. ADA Library & Archives; 2025. Available from: https://www.ada.org/resources/ada-library/oral-health-topics/dental-erosion. Accessed 25 June 2026.

[9] Jensdottir T, Holbrook P, Nauntofte B, Buchwald C, Bardow A. Immediate erosive potential of cola drinks and orange juices. J Dent Res. 2006;85(3):226–30. 10.1177/154405910608500304. 16498068 10.1177/154405910608500304

[10] Saads Carvalho T, Lussi A. Chap. 9: Acidic Beverages and Foods Associated with Dental Erosion and Erosive Tooth Wear. Monogr Oral Sci. 2020;28:91–8. 10.1159/000455376. 31940633 10.1159/000455376

[11] Fernández CE, Brandao ACS, Bícego-Pereira EC, Del Bel Cury AA, Cury JA, Tenuta LMA. Effect of pH and titratable acidity on enamel and dentine erosion. Clin Oral Investig. 2022;26(9):5867–73. 10.1007/s00784-022-04544-4. 35588021 10.1007/s00784-022-04544-4

[12] Tenuta LMA, Fernández CE, Brandão ACS, Cury JA. Titratable acidity of beverages influences salivary pH recovery. Brazilian Oral Res. 2015;29(1):1–6. 10.1590/1807-3107bor-2015.vol29.0032. 10.1590/1807-3107BOR-2015.vol29.0032 25715032

[13] Maguire A, Rugg-Gunn A. Xylitol and caries prevention—is it a magic bullet? Br Dent J. 2003;194(8):429–36. 10.1038/sj.bdj.4810022. 12778091 10.1038/sj.bdj.4810022

[14] Agrawal R, Trivedi S, Barodia Z. An Analysis of pH and Sugar Content of Commonly Prescribed Pediatric Liquid Medications: The Current Indian Scenario. J Pediatr Pharmacol Ther. 2024;29(4):354–8. 10.5863/1551-6776-29.4.354. 39144383 10.5863/1551-6776-29.4.354 PMC11321808

[15] Howard J, Dave M, Reynolds L, Westhead D, Barry S. The bitter truth regarding sugary medicines in children. BDJ Team. 2025;12(3):116–20. 10.1038/s41407-025-2926-x.

[16] Rouaz K, Chiclana-Rodríguez B, Nardi-Ricart A, Suñé-Pou M, Mercadé-Frutos D, Suñé-Negre JM, Pérez-Lozano P, García-Montoya E. Excipients in the paediatric population: a review. Pharmaceutics. 2021;13(3):387. 10.3390/pharmaceutics13030387. 10.3390/pharmaceutics13030387 PMC8000418 33805830

[17] Fatih MT, Mahmood MK, Kurda HA, Tassery H, Lan R, Tardivo D, Abdulghafor MA. Pediatric Liquid Medications and Dental Caries: A Narrative Review. Health Sci Rep. 2025;8(7):e71115. 10.1002/hsr2.71115. 40704323 10.1002/hsr2.71115 PMC12284424

[18] Nik-Hussein NN, Razak IA, Karim MN. An analysis of sugar content of commonly used pediatric liquid medicines–its relevance to dentistry. Singap Dent J. 1988;13(1):24–6. 3154999

[19] Babu KG, Doddamani GM, Naik LK, Jagadeesh K. Pediatric liquid medicaments–are they cariogenic? An in vitro study. J Int Soc Prev community dentistry. 2014;4(2):108–12. 10.4103/2231-0762.137637. 10.4103/2231-0762.137637 PMC4170542 25254195

[20] Elkhodary H, Alzughaibi O, Farsi D, Bastawy H, Bayoumi R, Felemban O. The impact of long-term use of pediatric liquid medications on primary tooth enamel: an in vitro study. J Clin Pediatr Dentistry. 2025;49(5):119–28. 10.22514/jocpd.2025.106.

[21] Al Humaid J. Sweetener content and cariogenic potential of pediatric oral medications: A literature. Int J Health Sci (Qassim). 2018;12(3):75–82. 29896075 PMC5969777

[22] Negrini TdC, Ren Z, Miao Y, Kim D, Simon-Soro Á, Liu Y, Koo H, Arthur RA. Dietary sugars modulate bacterial-fungal interactions in saliva and inter-kingdom biofilm formation on apatitic surface. Front Cell Infect Microbiol. 2022;12:993640. 10.3389/fcimb.2022.993640. 10.3389/fcimb.2022.993640 PMC9681999 36439211

[23] AlKanderi S, AlFreeh M, Bhardwaj RG, Karched M. Sugar Substitute Stevia Inhibits Biofilm Formation, Exopolysaccharide Production, and Downregulates the Expression of Streptococcal Genes Involved in Exopolysaccharide Synthesis. Dentistry J. 2023;11(12):267. 10.3390/dj11120267. 10.3390/dj11120267 PMC10742993 38132405

[24] World Health Organization. Guideline: sugars intake for adults and children. Geneva: World Health Organization; 2015. Available from: https://www.who.int/publications/i/item/9789241549028. Accessed 26 June 2026. 25905159

[25] Moynihan P. Sugars and Dental Caries: Evidence for Setting a Recommended Threshold for Intake. Adv Nutr. 2016;7(1):149–56. 10.3945/an.115.009365. 26773022 10.3945/an.115.009365 PMC4717883

[26] Baghlaf K, Muirhead V, Moynihan P, Weston-Price S, Pine C. Free Sugars Consumption around Bedtime and Dental Caries in Children: A Systematic Review. JDR Clin Translational Res. 2018;3:238008441774921. 10.1177/2380084417749215. 10.1177/2380084417749215 30931774

[27] Lussi A, Jaeggi T. Erosion—diagnosis and risk factors. Clin Oral Invest. 2008;12(Suppl 1):5–13. 10.1007/s00784-007-0179-z. 10.1007/s00784-007-0179-z PMC2238777 18228059

[28] Cavalcanti AL, Sousa RI, Clementino MA, Vieira FF, Cavalcanti CL, Xavier AF. In vitro analysis of the cariogenic and erosive potential of paediatric antitussive liquid oral medications. Tanzan J Health Res. 2012;14(2). 10.4314/thrb.v14i2.7. 26591735

[29] Singana T, Suma NK. An In Vitro Assessment of Cariogenic and Erosive Potential of Pediatric Liquid Medicaments on Primary Teeth: A Comparative Study. Int J Clin Pediatr Dent. 2020;13(6):595–9. 10.5005/jp-journals-10005-1824. 33976481 10.5005/jp-journals-10005-1824 PMC8060941

[30] Nankar M, Walimbe H, Bijle MNA, Kontham U, Kamath A, Muchandi S. Comparative evaluation of cariogenic and erosive potential of commonly prescribed pediatric liquid medicaments: an in vitro study. J Contemp Dent Pract. 2014;15(1):20–5. 10.5005/jp-journals-10024-1481. 24939259 10.5005/jp-journals-10024-1481

[31] Maguire A, Baqir W, Nunn JH. Are sugars-free medicines more erosive than sugars‐containing medicines? An in vitro study of paediatric medicines with prolonged oral clearance used regularly and long‐term by children. Int J Pediatr Dent. 2007;17(4):231–8. 10.1111/j.1365-263X.2007.00826.x. 10.1111/j.1365-263X.2007.00826.x 17559449

[32] Bolgül B, Arıkan R, Peker O. Evaluation of cariogenic and erosive potentials of pediatric liquid medicines. Selcuk Dent J. 2024;11(2):211–7. 10.15311/selcukdentj.1387296.

[33] Jung EH, Jun MK. Evaluation of the erosive and cariogenic potential of over-the-counter pediatric liquid analgesics and antipyretics. Children. 2021;8(7):611. 10.3390/children8070611. 10.3390/children8070611 PMC8306722 34356590

[34] European Medicines Agency. Reflection paper on formulations of choice for the paediatric population (EMEA/CHMP/PEG/194810/2005). London: European Medicines Agency; 2006. Available from: https://www.ema.europa.eu/en/formulations-choice-paediatric-population-scientific-guideline. Accessed 26 June 2026.

[35] Salunke S, Brandys B, Giacoia G, Tuleu C. The STEP (Safety and Toxicity of Excipients for Paediatrics) database: part 2–the pilot version. Int J Pharm. 2013;457(1):310–22. 10.1016/j.ijpharm.2013.09.013. 24070789 10.1016/j.ijpharm.2013.09.013

[36] Belayneh A, Tadese E, Molla F. Safety and Biopharmaceutical Challenges of Excipients in Off-Label Pediatric Formulations. Int J Gen Med. 2020;13:1051–66. 10.2147/ijgm.S280330. 33204140 10.2147/IJGM.S280330 PMC7667588

[37] Moynihan PJ, Kelly SAM. Effect on Caries of Restricting Sugars Intake:Systematic Review to Inform WHO Guidelines. J Dent Res. 2014;93(1):8–18. 10.1177/0022034513508954. 24323509 10.1177/0022034513508954 PMC3872848

[38] Newton PN, Green MD, Fernández FM. Impact of poor-quality medicines in the ‘developing’world. Trends Pharmacol Sci. 2010;31(3):99–101. 10.1016/j.tips.2009.11.005. 20117849 10.1016/j.tips.2009.11.005 PMC2845817

[39] Almuzaini T, Choonara I, Sammons H. Substandard and counterfeit medicines: a systematic review of the literature. BMJ open. 2013;3(8):e002923. 10.1136/bmjopen-2013-002923. 23955188 10.1136/bmjopen-2013-002923 PMC3752049

[40] Kaplan WA, Cellini CM, Eghan K, Pilz K, Harrison D, Wirtz VJ. Contracting retail pharmacies as a source of essential medicines for public sector clients in low- and middle-income countries: a scoping review of key considerations, challenges, and opportunities. J Pharm Policy Pract. 2023;16(1):60. 10.1186/s40545-023-00557-w. 37131256 10.1186/s40545-023-00557-w PMC10153779

[41] Birkhed D. Sugar content, acidity and effect on plaque pH of fruit juices, fruit drinks, carbonated beverages and sport drinks. Caries Res. 1984;18(2):120–7. 10.1159/000260759. 6583004 10.1159/000260759

[42] Valinoti AC, Costa A Jr, Pereira de Sousa V, Fonseca-Gonçalves A, Maia LC. Are Pediatric Antibiotic Formulations Potentials Risk Factors for Dental Caries and Dental Erosion? Open Dentistry J. 2016;10. 10.2174/1874210601610010420. 10.2174/1874210601610010420 PMC4995533 27583053

[43] Featherstone JD. The science and practice of caries prevention. J Am Dent association. 2000;131(7):887–99. 10.14219/jada.archive.2000.0307. 10.14219/jada.archive.2000.0307 10916327

[44] Zero DT. Etiology of dental erosion–extrinsic factors. Eur J Oral Sci. 1996;104(2):162–77. 10.1111/j.1600-0722.1996.tb00065.x. 8804884 10.1111/j.1600-0722.1996.tb00065.x

[45] Sheiham A, James WPT. Diet and Dental Caries:The Pivotal Role of Free Sugars Reemphasized. J Dent Res. 2015;94(10):1341–7. 10.1177/0022034515590377. 26261186 10.1177/0022034515590377

[46] Lussi A, Schlueter N, Rakhmatullina E, Ganss C. Dental Erosion – An Overview with Emphasis on Chemical and Histopathological Aspects. Caries Res. 2011;45(Suppl 1):2–12. 10.1159/000325915. 21625128 10.1159/000325915

[47] Harris DC. Quantitative chemical analysis. Macmillan; 2010.

[48] AOAC. Official Methods of Analysis (Lane–Eynon method). Maryland: AOAC International 2019. doi.

[49] Ranganna S. Handbook of analysis and quality control for fruit and vegetable products. Tata McGraw-Hill Education; 1986.

[50] Kim H-Y. Analysis of variance (ANOVA) comparing means of more than two groups. Restor Dent Endod. 2014;39(1):74–7. 10.5395/rde.2014.39.1.74. 24516834 10.5395/rde.2014.39.1.74 PMC3916511

[51] Barbour M, Lussi A. Erosion in relation to nutrition and the environment. Erosive Tooth Wear: Diagnos Th. 2012;25:143–54. 10.1159/000359941. 10.1159/000359941 24993263

[52] Moynihan P, Petersen PE. Diet, nutrition and the prevention of dental diseases. Public Health Nutr. 2004;7(1a):201–26. 10.1079/phn2003589. 14972061 10.1079/phn2003589

[53] Xavier AFC, Moura EF, Azevedo WF, Vieira FF, Abreu MH, Cavalcanti AL. Erosive and cariogenicity potential of pediatric drugs: study of physicochemical parameters. BMC Oral Health. 2013;13(1):1–7. 10.1186/1472-6831-13-71. 24325544 10.1186/1472-6831-13-71 PMC4029480

[54] Siddiq H, Pentapati KC, Shenoy R, Velayutham A, Acharya S. Evaluation of sugar content and erosive potential of the commonly prescribed liquid oral medications. Pesquisa Brasileira em Odontopediatria e Clínica Integrada. 2020;20:e5025. 10.1590/pboci.2020.023.

[55] Gupta M, Panda S. Cariogenic Potential of the commonly Prescribed Pediatric Liquid Medicaments in Kingdom of Saudi Arabia: An in vitro Study. J Contemp Dent Pract. 2017;18(4):307–11. 10.5005/jp-journals-10024-2036. 28349909 10.5005/jp-journals-10024-2036

[56] Mahmoud EF, Omar OM. Erosive and cariogenic potential of various pediatric liquid medicaments on primary tooth enamel: A SEM study. Dent Med Probl. 2018;55(3):247–54. 10.17219/dmp/91539. 30328301 10.17219/dmp/91539

[57] Passos IA, Sampaio FC, Martínez CR, Freitas CHSM. Sucrose concentration and pH in liquid oral pediatric medicines of long-term use for children. Rev Panam Salud Publica. 2010;27:132–7. 10.1590/s1020-49892010000200007. 20339617 10.1590/s1020-49892010000200007

[58] Subramaniam P, Nandan N. Cariogenic potential of pediatric liquid medicaments-An in vitro study. J Clin Pediatr Dentistry. 2012;36(4):357–62. 10.17796/jcpd.36.4.nt11584612462t84. 10.17796/jcpd.36.4.nt11584612462t84 23019832

[59] Cavalcanti AL, Fernandes LV, Barbosa AS, Vieira FF. pH, titratable acidity and total soluble solid content of pediatric antitussive medicines. Acta Stomatol Croatica: Int J oral Sci Dent Med. 2008;42(2):164–70.

[60] Young W, Khan F. Sites of dental erosion are saliva-dependent. J Rehabil. 2002;29(1):35–43. 10.1046/j.1365-2842.2002.00808.x. 10.1046/j.1365-2842.2002.00808.x 11844030

[61] Touger-Decker R, van Loveren C. Sugars and dental caries. Am J Clin Nutr. 2003;78(4):s881–92. 10.1093/ajcn/78.4.881S. 10.1093/ajcn/78.4.881S 14522753

[62] Congiu G, Campus G, Lugliè PF. Early childhood caries (ECC) prevalence and background factors: a review. Oral Health Prev Dent. 2014;12(1):71–6. 10.3290/j.ohpd.a31216. 24619785 10.3290/j.ohpd.a31216

[63] Santos FGd, Pereira AMBC, Farias IAP, Passos, TAd. Oliveira AFBd: Erosive Effect of Long-Term Liquid Oral Pediatric Medicines on Permanent Tooth Enamel. Pesquisa Brasileira em Odontopediatria e Clínica Integrada. 2025;25:e230119. 10.1590/pboci.2025.068.

[64] Bigeard L. The role of medication and sugars in pediatric dental patients. Dent Clin North Am. 2000;44(3):443–56. 10.1016/S0011-8532(22)01740-2. 10925767

[65] Taji S, Seow W. A literature review of dental erosion in children. Aust Dent J. 2010;55(4):358–67. 10.1111/j.1834-7819.2010.01255.x. 21133936 10.1111/j.1834-7819.2010.01255.x

[66] Valinoti AC, da Silva Pierro VS, Da Silva EM, Maia LC. In vitro alterations in dental enamel exposed to acidic medicines. Int J Pediatr Dent. 2011;21(2):141–50. 10.1111/j.1365-263X.2010.01104.x. 10.1111/j.1365-263X.2010.01104.x 20961343

[67] Zero D. Sugars–the arch criminal? Caries Res. 2004;38(3):277–85. 10.1159/000077767. 15153701 10.1159/000077767

[68] ICH E11 (R1.) guideline on clinical investigation of medicinal products in the pediatric population.

[69] European Medicines Agency. Reflection paper on formulations of choice for the paediatric population. Report No. EMEA/CHMP/PEG/194810/2005. London: European Medicines Agency; 2006. Available from: https://www.ema.europa.eu/en/documents/scientific-guideline/reflection-paper-formulations-choice-paediatric-population_en.pdf. Accessed 26 June 2026.

[70] European Medicines Agency. Draft guideline on pharmaceutical development of medicines for paediatric use. Report No. EMA/CHMP/QWP/180157/2011. London: European Medicines Agency; 2011. Available from: https://www.ema.europa.eu/en/documents/scientific-guideline/draft-guideline-pharmaceutical-development-medicines-paediatric-use_en.pdf-0. Accessed 26 June 2026.

[71] Bradley MB, Kinirons MJ. Choice of sugar-free medicines by a sample of dentists, doctors and pharmacists in Northern Ireland: the views of parents and health professionals. Community Dent Health. 1998;15(2):105–8. 9793227

