# Supplementary material for: Physicochemical evaluation of pediatric liquid medicines in Ghana: implications for cariogenicity and erosive potential
Source: BMC Oral Health. 2026 Jun 27;26:1426. doi: 10.1186/s12903-026-09011-0 (PMC13463936; doi:10.1186/s12903-026-09011-0)
Supplement: Supplementary file 1 [file 12903_2026_9011_MOESM1_ESM.docx]

**Supplementary Table**

| **Table S1.** Characteristics of pediatric liquid medicines included in the study, showing sample identification codes, therapeutic class, generic composition, origin, dosage form, and labelled instructions for use. | | | | | |
| --- | --- | --- | --- | --- | --- |
| **Sample ID** | **Therapeutic Class** | **Generic Name** | **Origin** | **Dosage Form** | **Labelled Instructions for Use** |
| AN1 | Analgesics | Paracetamol | Foreign | Syrup | As directed by physician / per label |
| AN2 | Analgesics | Paracetamol | Foreign | Syrup | As directed by physician / per label |
| AN3 | Analgesics | Paracetamol | Local | Syrup | As directed by physician / per label |
| AN4 | Analgesics | Paracetamol | Local | Syrup | As directed by physician / per label |
| AN5 | Analgesics | Paracetamol | Local | Syrup | As directed by physician / per label |
| AT1 | Antitussives | Dextromethorphan,  Chlorpheniramine maleate | Foreign | Syrup | As directed by physician / per label |
| AT2 | Antitussives | Dextromethorphan,  Guaifenesin | Foreign | Syrup | As directed by physician / per label |
| AT3 | Antitussives | Chlorpheniramine maleate,  Guaifenesin | Local | Syrup | As directed by physician / per label |
| AT4 | Antitussives | Ipecacuanha Liquid Extract,  Squill Vinegar Extract | Local | Syrup | As directed by physician / per label |
| AT5 | Antitussives | Diphenhydramine,  Ammonium chloride | Local | Syrup | As directed by physician / per label |
| AH1 | Antihistamines | Chlorphenamine | Foreign | Syrup | As directed by physician / per label |
| AH2 | Antihistamines | Cetirizine | Foreign | Syrup | As directed by physician / per label |
| AH3 | Antihistamines | Cetirizine | Local | Syrup | As directed by physician / per label |
| AH4 | Antihistamines | Cetirizine | Local | Syrup | As directed by physician / per label |
| AH5 | Antihistamines | Cetirizine | Local | Syrup | As directed by physician / per label |
| NS1 | NSAID | Ibuprofen | Foreign | Suspension | As directed by physician / per label |
| NS2 | NSAID | Ibuprofen | Foreign | Suspension | As directed by physician / per label |
| NS3 | NSAID | Ibuprofen | Local | Suspension | As directed by physician / per label |
| NS4 | NSAID | Ibuprofen | Local | Suspension | As directed by physician / per label |
| NS5 | NSAID | Ibuprofen | Local | Suspension | As directed by physician / per label |
| AM1 | Antimalarials | Artemether/Lumefantrine | Foreign | Suspension | As directed by physician / per label |
| AM2 | Antimalarials | Artemether/Lumefantrine | Foreign | Suspension | As directed by physician / per label |
| AM3 | Antimalarials | Artemether/Lumefantrine | Local | Suspension | As directed by physician / per label |
| AM4 | Antimalarials | Artemether/Lumefantrine | Local | Suspension | As directed by physician / per label |
| AM5 | Antimalarials | Artemether/Lumefantrine | Local | Suspension | As directed by physician / per label |
| AB1 | Antibiotics | Amoxicillin/Clavulanic Acid | Foreign | Suspension | As directed by physician / per label |
| AB2 | Antibiotics | Amoxicillin/Clavulanic Acid | Foreign | Suspension | As directed by physician / per label |
| AB3 | Antibiotics | Metronidazole | Local | Suspension | As directed by physician / per label |
| AB4 | Antibiotics | Metronidazole | Local | Suspension | As directed by physician / per label |
| AB5 | Antibiotics | Flucloxacillin | Local | Suspension | As directed by physician / per label |
| **Abbreviations:** AN, analgesics; AT, antitussives; AH, antihistamines; NS, non-steroidal anti-inflammatory drugs; AM, antimalarials; AB, antibiotics. | | | | | |
